# Multigene Molecular Phylogeny and Biogeographic Diversification of the Earth Tongue Fungi in the Genera *Cudonia* and *Spathularia* (Rhytismatales, Ascomycota)

**DOI:** 10.1371/journal.pone.0103457

**Published:** 2014-08-01

**Authors:** Zai-Wei Ge, Zhu L. Yang, Donald H. Pfister, Matteo Carbone, Tolgor Bau, Matthew E. Smith

**Affiliations:** 1 Key Laboratory for Plant Diversity and Biogeography of East Asia, Kunming Institute of Botany, Chinese Academy of Sciences, Kunming, Yunnan, China; 2 Harvard University Herbaria and Department of Organismic and Evolutionary Biology, Harvard University, Cambridge, Massachusetts, United States of America; 3 Department of Plant Pathology, University of Florida, Gainesville, Florida, United States of America; 4 Via Don Luigi Sturzo 173, Genova, Italy; 5 Institute of Mycology, Jilin Agriculture University, Changchun, Jilin, China; BiK-F Biodiversity and Climate Research Center, Germany

## Abstract

The family Cudoniaceae (Rhytismatales, Ascomycota) was erected to accommodate the “earth tongue fungi” in the genera *Cudonia* and *Spathularia*. There have been no recent taxonomic studies of these genera, and the evolutionary relationships within and among these fungi are largely unknown. Here we explore the molecular phylogenetic relationships within *Cudonia* and *Spathularia* using maximum likelihood and Bayesian inference analyses based on 111 collections from across the Northern Hemisphere. Phylogenies based on the combined data from ITS, nrLSU, *rpb2* and *tef-1α* sequences support the monophyly of three main clades, the /flavida, /velutipes, and /cudonia clades. The genus *Cudonia* and the family Cudoniaceae are supported as monophyletic groups, while the genus *Spathularia* is not monophyletic. Although Cudoniaceae is monophyletic, our analyses agree with previous studies that this family is nested within the Rhytismataceae. Our phylogenetic analyses circumscribes 32 species-level clades, including the putative recognition of 23 undescribed phylogenetic species. Our molecular phylogeny also revealed an unexpectedly high species diversity of *Cudonia* and *Spathularia* in eastern Asia, with 16 (out of 21) species-level clades of *Cudonia* and 8 (out of 11) species-level clades of *Spathularia*. We estimate that the divergence time of the *Cudoniaceae* was in the Paleogene approximately 28 Million years ago (Mya) and that the ancestral area for this group of fungi was in Eastern Asia based on the current data. We hypothesize that the large-scale geological and climatic events in Oligocene (e.g. the global cooling and the uplift of the Tibetan plateau) may have triggered evolutionary radiations in this group of fungi in East Asia. This work provides a foundation for future studies on the phylogeny, diversity, and evolution of *Cudonia* and *Spathularia* and highlights the need for more molecular studies on collections from Europe and North America.

## Introduction

Fungi are the principal degraders of biomass in terrestrial ecosystems [1], [2]. As decomposers of organic matter, they form a significant component of forest ecosystems. However, compared to the 5.1 million estimated fungal species [3], our current understanding of fungal evolutionary diversity is limited [4], [5] with an estimated 100,000 species currently recognized [3]. This lack of basic information on diversity has significantly hampered our interpretations of biogeographic patterns in fungi [6], [7]. This is particularly true for saprotrophic fungi, such as the Earth Tongue mushrooms, which are ephemeral, easily overlooked on the forest floor, and have no commercial value as food.

**Figure 1:**
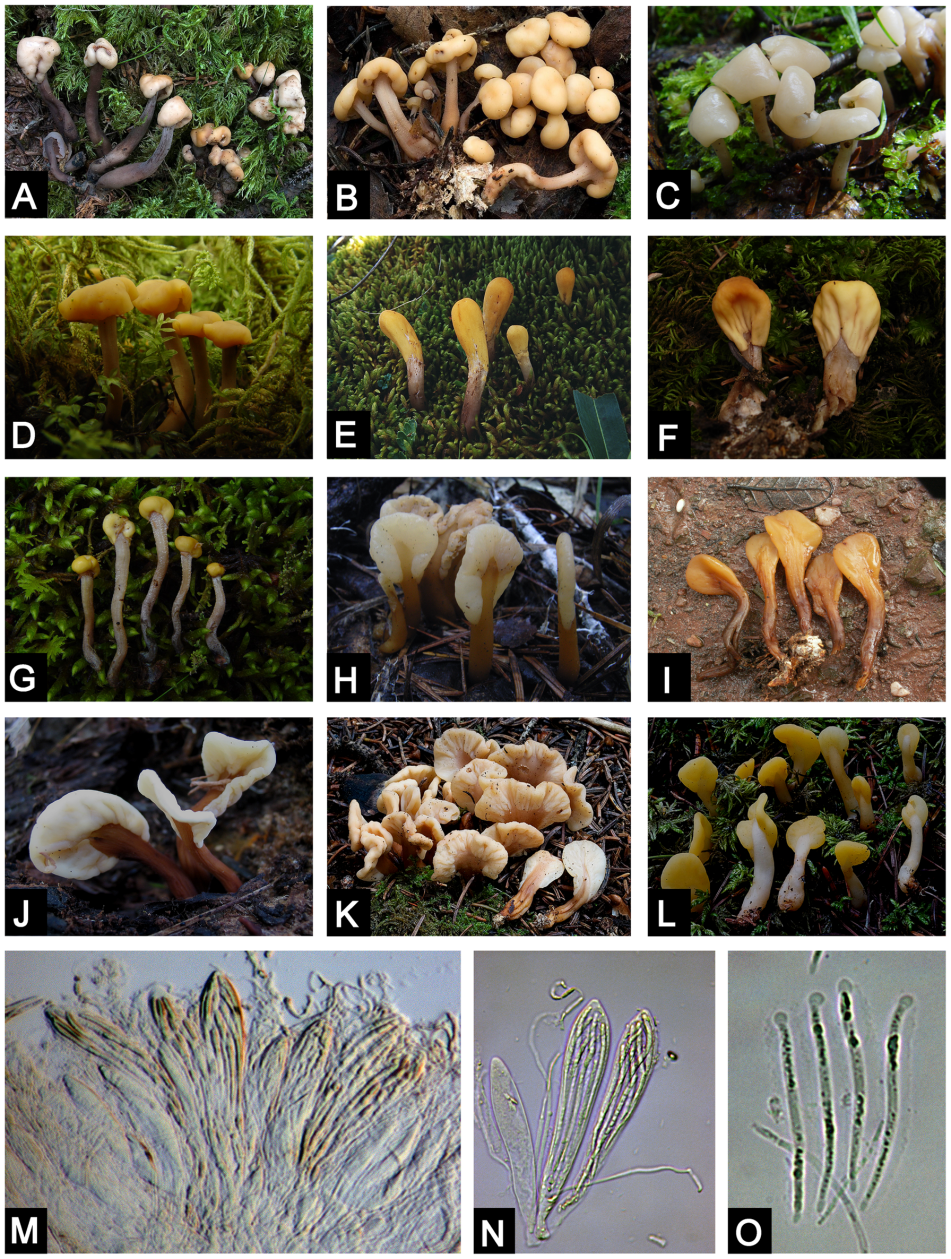
Macroscopic and microscopic morphological features of *Cudonia* and *Spathularia* species. A. *Cudonia circinans* (M. Carbone 313); B. *Cudonia confusa* (M. Carbone 314); C. *Cudonia* sp1 (Z.-W. Ge 729); D. *Cudonia lutea* (Z.-W. Ge 1634); E. *Cudonia* sp10 (Z. L. Yang 4297); F. *Cudonia* sp9 (X. H. Wang 2324); G. *Cudonia* sp12 (Z.-W. Ge 829); H. *Spathularia* sp5 (Z. L. Yang 5385); I. *Spathularia* sp4 (L. P. Tang 252); J. *Spathularia velutipes* (Z.-W. Ge 2232); K. *Spathularia rufa* (M. Carbone 309); L. *Spathularia flavida* (M. Carbone 313); M. Hymenium of *Spathularia flavida* (Z.-W. Ge 3348); N. Asci and paraphyses of *Spathularia flavida* (Z.-W. Ge 3348); O. Ascospores of *Spathularia flavida* (Z.-W. Ge 3348).

The family Cudoniaceae (Rhytismatales, Ascomycotina) was erected by P. F. Cannon to accommodate the genera *Cudonia* Fr. and *Spathularia* Pers. [8]. Members of this family are usually referred to in fungi guidebooks as “earth tongues” or “fairy fans” because of their shapes (Fig. 1). These fungi form small fruiting bodies that have a flattened to club-shaped apex. *Cudonia* and *Spathularia* are restricted to the Northern Hemisphere with highest species diversity in temperate habitats and only a few species reported from subtropical areas. However, species of *Cudonia* and *Spathularia* have not been recovered in molecular surveys as root endophytes or mycorrhizas and are thus presumed to be soil and leaf litter saprotrophs.

*Cudonia* differs from *Spathularia* in that the *Spathularia* species have flattened fruiting bodies (e.g. like a spatula) whereas *Cudonia* species have rounded and club-shaped fruiting bodies (Fig. 1). However, the micromorphology of these two genera is similar: both genera have curved paraphyses, club-shaped asci, and long, thin, hyaline ascospores that are enveloped in gelatinous sheaths [8], [9]. These characters are also shared by many species of Rhytismataceae Chevall.

Members of *Cudonia* and *Spathularia* differ morphologically from other species in the family Rhytismataceae, such as *Rhytisma* Fr. species that cause “tar spot” on leaves [10], *Coccomyces* De Not. species that cause “leaf blights” [11], and some *Lophodermium* Chevall. species that cause “needle cast” diseases on conifers [12]. These members of Rhytismatales M. E. Barr. ex Minter have inconspicuous fruiting bodies where the spore-bearing hymenium is covered by tissue (at least during early development). Although *Cudonia* and *Spathularia* have distinct fruit bodies, with a stipe and spore bearing hymenium, the hymenium is covered in early development by a thin veil [13]-[17]. This veil can be seen in mature specimens of *Cudonia* and *Spathularia* as a skirt-like structure at the margin of the hymenium and this feature may be homologous to the covering layer that is present in some Rhytismataceae species (e.g. *Rhytisma* spp. [10]).

Because of the unique morphology of *Cudonia* and *Spathularia* species, there have been a large number of taxonomic studies of this group in Europe [18]-[20], North America [14]-[16], [21], and Asia [9], [22], [23]. *Cudonia* and *Spathularia* were traditionally placed in the order Helotiales Nannf. ex Korf & Lizon, with *Cudonia* in the Leotiaceae Corda and *Spathularia* in Geoglossaceae Corda [24]. However, the Geoglossaceae has recently been redefined and placed it in its own order (Geoglossales) and class (Geoglossomycetes) [25]. When P. F. Cannon erected Cudoniaceae with *Cudonia* as the type genus, the group was considered to belong in Helotiales [8] but was later transferred to Rhytismatales [26], [27].

Although the taxonomy of *Cudonia* and *Spathularia* has received ample attention in the past, this group has rarely been examined using molecular tools. Most studies that have included sequences of *Cudonia* and *Spathularia* have been focused on family-level, order-level, or class-level phylogenetic relationships, and thus have incorporated only a few species from these genera. Phylogenetic analyses based on the 18S nuc-SSU nrDNA [28]-[31] showed that *C. circinans* and *S. flavida* formed a monophyletic clade. This relationship was later confirmed based on the ITS and 28S nrLSU [9], [17], [32] and other genes [33]-[35]. Although only a few species of *Cudonia* and *Spathularia* included, the most recent findings based on nrLSU and mtSSU indicate an affinity between *Cudonia, Spathularia,* and members of *Coccomyces*, *Colpoma* Wallr., and *Tryblidiopsis* P. Karst. (Rhytismataceae, Rhytismatales) [27], suggesting that the Cudoniaceae arose from within Rhytismataceae.

Until now, only one phylogenetic analysis [9] has examined relationships within *Cudonia* and *Spathularia*. The combined ITS-nrLSU analysis recovered both genera as monophyletic but with low bootstrap support. However, this study was based on a small number of specimens from a few species and the ribosomal genes were insufficient to adequately resolve the relationships within the group. Thus, there are still many open questions about the relationships both within and between *Cudonia* and *Spathularia*. For instance, no studies have examined this group based on a geographically and phylogenetically broad sampling of specimens, no studies have used a multi-gene phylogeny to study this group, and no studies have inferred biogeographic or morphological evolutionary patterns within *Cudonia* and *Spathularia.* In recent years, multi-gene datasets have proved successful for species delimitation and for inferring robust phylogenies of major fungal clades [36]. Well-resolved phylogenies have subsequently facilitated biogeographic studies of several widespread and ecologically important fungal groups [37]-[42].

This study investigates the species diversity, biogeography, and phylogenetic relationships within and between *Cudonia* and *Spathularia*. To accomplish this goal we have performed maximum likelihood (ML) and Bayesian inference (BI) phylogenetic analyses based on four DNA regions: the nuclear internal transcribed spacers (ITS), the nuclear large subunit ribosomal DNA (nrLSU), the second largest subunit of RNA polymerase II (*rpb2*), and elongation factor 1-α (*tef-1α*). We also performed Bayesian molecular clock analysis to estimate evolutionary divergence times for *Cudonia* and *Spathularia* and we used Likelihood analysis of geographic range evolution to hypothesize the putative center of origin for this group. The specific aims of this study were: (i) to examine genetic differentiation and species diversity among a global sampling of *Cudonia* and *Spathularia* specimens; (ii) to infer the phylogenetic relationships within this group using ribosomal DNA sequences (ITS and nrLSU) and two single-copy nuclear genes (*rpb2* and *tef-1α*); (iii) to infer the biogeography of *Cudonia* and *Spathularia* and to elucidate both its center of diversity and the main biogeographic events; and (iv) to discuss the morphological evolution that occurred during their radiation in the Northern Hemisphere.

## Materials and Methods

### Taxon sampling

We obtained 111 collections of *Cudonia* and *Spathularia* from throughout the Northern Hemisphere (Table S1). Most collections (105) were obtained over the last decade where *Cudonia* and *Spathularia* are known to occur; Asia (China), Europe (Finland, Switzerland) and North America (Canada, United States). The remaining six specimens were obtained from the fungal herbaria at Jilin Agricultural University (HMJAU) (2 specimens) and the University of Tennessee (TENN) (4 specimens). Since species within both *Cudonia* and *Spathularia* have no protected status, no specific permits were required for collecting these fungi. Voucher specimens are housed in the Cryptogamic Herbarium of the Kunming Institute of Botany, Chinese Academy of Sciences (HKAS) and the Farlow Herbarium of Harvard University (FH).

### DNA extraction, PCR and Sequencing

DNA was extracted from approximately 20 mg of dried fruiting body tissue using the DNeasy Plant Mini Kit (Qiagen, Maryland, US) according to the manufacturer's recommendations or using a modified CTAB method [43].

PCR reactions were performed on an ABI 2720 Thermal Cycler (Applied Biosystems, Foster City, CA, USA) or an Eppendorf Master Cycler (Eppendorf, Netheler-Hinz, Hamburg, Germany), using Biomed taq and their suggested protocols (Biomed, Beijing 100097, China).

We used published primers to PCR-amplify fragments from the ITS, nrLSU, *rpb2* and *tef-1α* DNA regions: ITS1F/ITS4 for ITS [44], [45], LR0R/LR5 for nrLSU [46], 983F/1567R for *tef-1α* [47], and *RPB2*-6F and *RPB2*-7R for *rpb2* [48]. The *rpb*2 region was difficult to amplify with published primers, so for difficult samples we designed a new primer pair (RPB2_6Cudf: 5′- TCAGGCTTGTGGTCTGGT -3′; RPB2-7Cudr: 5′- GGGAAGGGAATGATGGAT -3′) using the online software package Primer3 [49].

For PCR amplification, we used published thermocycler conditions [50] except that annealing temperatures were optimized for each gene region: 53°C for ITS, 50°C for nrLSU, 60°C for *rpb2*, or 55°C for *tef-1α*. Amplicons were electrophoresed in 1% agarose gels stained with SYBR Green I (Molecular Probes, Eugene, OR, USA) or Gel View (BioTeke Corporation, Beijing, China) and then visualized under UV light. PCR products were purified using a QIAquick PCR purification kit (Qiagen Science, USA) or EXO and SAP enzymes [51]. When multiple amplicons were present, we excised amplicons of the appropriate size using a Gel Extraction & PCR Purification Combo Kit (Spin-column) (Bioteke, Beijing, China; Qiagen Science, USA).

Sequencing was performed with the same PCR primers using the Big Dye Sequencing Kit v.3.1 on an ABI-3730-XL DNA Analyzer (Applied Biosystems, Foster City, CA, USA) or sent to Shanghai Sangon Biological Engineering Technology and Service Co. for sequencing. For samples that failed in direct sequencing, PCR products were gel purified and then cloned in the TakaRa pMD18-T Vector. Two to three clones per amplicon were sequenced. Sequence chromatograms were compiled with Sequencher v4.1.4 (Gene Codes Corp., Ann Arbor, MI, USA) or Seqman (DNA STAR Package; DNAStar, Madison, WI, USA). All sequences have been deposited in GenBank (Table S1).

### Data matrices and phylogenetic analyses

*Coccomyces dentatus* (J.C. Schmidt &Kunze) Sacc. and *Tryblidiopsis pinastri* (Pers.) P. Karst. (Rhytismatales) were selected as outgroups for phylogenetic analyses except for the ITS dataset. These genera are considered close relatives of *Cudonia* and *Spathularia* by previous studies [27], [31], [52], and our preliminary analysis, including diverse Rhytismatales and Helotiales, verified that these taxa are appropriate outgroups.

After adding *Cudonia* and *Spathularia* sequences from GenBank (16 ITS, 17 nrLSU, 2 *rpb2*, and 1 *tef-1α* – see Tables S2 and S3) with sequences from our 111 collections, phylogenetic reconstructions were conducted to identify well-supported clades of *Cudonia* and *Spathularia*. Alignments were performed for each gene (ITS, nrLSU, *rpb2* and *tef-1α*) using Mafft v6.8 [53] with manual improvements including trim the ends and adjust the obviously misaligned base pairs in Bioedit v7.0.9 [54]. The alignments (sites excluded from the analyses listed after the alignment file) have been submitted to TreeBase with submission number S15957 (http://purl.org/phylo/treebase/phylows/study/TB2:S15957). In the 127 specimens ITS alignment, 75 out of 587 sites have been excluded for analyses, most of these sites are gaps due to sequences downloaded from GenBank (particularly *Cudonia monticola* and *Spathularia* sp.). These gaps are obvious introns that are clearly visible and easy to interpret (Data S1). The sites excluded from analyses are: 11, 35, 61, 69-70, 78, 82-85, 91, 106, 117, 123-125, 144-158, 167, 180-181, 193-194, 211-215, 224-225, 229, 392-393, 410-414, 440-447, 482-483, 501-503, 517, 524, 544, 554-560, and 567.

A reciprocal 70% bootstrap support approach [55] was used to compare the tree topologies from individual genes. The result revealed there is no significant incongruence between the data sets, so the ITS, nrLSU, *rpb2*, *tef-1α* were concatenated for phylogenetic reconstruction.

Maximum Likelihood analyses were performed to infer phylogenetic relationships for each gene and for the combined ITS-nrLSU-*rpb2*-*tef-1α* dataset using RAxML version 7.2.3 [56]. Optimal substitution models for each dataset were determined using Akaike Information Criterion (AIC) implemented in MrModeltest v2.3 [57]: GTR+G+I for ITS, HKY+I+G for nrLSU, GTR+G for *rpb2*, SYM+I+G for *tef-1α*, and HKY+G+I for 5.8S. The GTR+G+I model of evolution was selected for the multi-gene dataset. The ML analyses were implemented using default settings with gaps treated as missing data and branch support was assessed through 1000 bootstrap partitions (BP) with the rapid bootstrap option.

Bayesian Inference (BI) analysis was conducted for the combined dataset using MrBayes 3.1.2 [58]. Bayesian posterior probabilities were determined twice by running one cold and three heated chains for fifty million generations using selected models, saving trees every 1000th generation. Burn-ins were determined by checking the likelihood trace plots in Tracer v1.5 (http://tree.bio.ed.ac.uk/software/tracer/) and subsequently discarded. The partitioned mixed model, which allows for model parameters estimated separately for each gene, was used in the combined analysis. Chain convergence was determined using Tracer v1.5 to ensure most of the effective sample size (ESS) values were above 200.

### Phylogenetic species determination

To circumscribe species within *Cudonia* and *Spathularia* that have multiple collections and sequences from multiple loci available, we applied the multilocus genealogical phylogenetic species recognition approach [59], [60]. A phylogenetic species is recognized when it satisfies either of two criteria: (1) a genealogical concordant group that is present in the majority of the single-locus genealogies or (2) a clade that is strongly supported by at least one single-locus genealogy and is not contradicted by any other locus. For putatively distinct taxa with only a single ITS nrDNA sequence, we calculated the genetic distance compared to the ITS nrDNA for all other recognized species to assess the likelihood that the organism with the unique sequence should be recognized as a distinct species [37], [61]. Intra- and inter-specific variation within species of *Cudonia* and *Spathularia* were separately calculated using ITS alignments. If the sequence variation between the putative new species and all other recognized species was higher than the variation among the known species, then the putative species was recognized as a separate taxon. To assist in species delimitation, barcode gaps between the infraspecific and interspecific divergence were also calculated using the Automatic Barcode Gap Discovery (ABGD) method [62].

### Diversification time estimates

To infer the “time to most recent common ancestor” (tMRCA), we used the secondary calibration strategy implemented by Renner [63]. Initially, to infer the stem node age of Cudoniaceae, we built a 5.8S-nrLSU-*rpb2*-*tef-1α* dataset that included 33 taxa from across major clades of Pezizomycotina [34], [64]. We then used this stem node age to calibrate the same node in a multi-gene (ITS-nrLSU-*rpb2*-*tef-1α*) analysis that included 2 taxa for calibration and 25 species of *Cudonia* and *Spathularia*.

The initial, phylogenetically broad analysis included representatives from most major clades within Pezizomycotina as well as representatives from Saccharomycotina, Taphrinomycotina, and Basidiomycota. *Rhizopus oryzae*, a member of Zygomycota, was chosen as the outgroup taxon (Table S2). Three calibration points were used for the initial analysis: the stem of Ascomycota (575 ± 37.5 Mya), the stem of Pezizomycotina (460 ± 30 Mya), and the crown of Pezizomycotina (360 ± 20 Mya). These values are the mean ranges of dates (500–650 Mya, 400-520 Mya, and 320-400 Mya respectively), with a standard deviation that produces a central 95% range of dates corresponding to those reported by Lücking et al. [65] for these nodes.

Both the initial and secondary tMRCA analyses were performed using BEAST v. 1.5.3 [66]. We used a Yule tree prior that assumes a constant lineage birth rate for each branch in the tree and we also used a relaxed lognormal molecular clock prior so that substitution rates were allowed to vary across branches. Priors of the calibration points were constrained to be normally distributed with a standard deviation as mentioned above.

For each analysis, two independent MCMC analyses were performed with parameter values sampled every 1,000 cycles over 50,000,000 MCMC steps. Convergence and acceptable mixing of the sampled parameters was verified using the program Tracer v. 1.5 [66]. After discarding the burn-in, the mean ± standard error and the 95% highest posterior density (HPD) interval for the divergence dates of the ancestral nodes were calculated from combined BEAST log files.

### Ancestral area reconstruction analysis

To reconstruct the possible ancestral areas for *Cudonia* and *Spathularia*, ML-based dispersal-extinction-cladogenesis module was performed using LAGRANGE [67]. We used our multi-gene ML phylogeny and the diversification time of *Cudonia* and *Spathularia* estimated by BEAST to reconstruct the possible historical distribution of these genera. For the analyses of range evolution, a species was considered endemic if it was restricted to Eurasia or North America. We determined the presence or absence of each species of *Cudonia* or *Spathularia* in each of these world regions: Asia (A), Europe (E), and North America (N).

## Results

### Phylogenetic analyses of *Cudonia* and *Spathularia* based on individual genes

We generated 372 new sequences from 111 collections of *Cudonia* and *Spathularia*, including 111 sequences of ITS, 94 of nrLSU, 74 of *rpb2*, and 93 of *tef-1α*.

The aligned ITS dataset consisted of 127 sequences and was 587 nucleotides in length. We excluded 75 ambiguously aligned sites that consisted of introns found only in the divergent GenBank sequences of *Cudonia monticola* and three sequences labeled as “Uncultured Ascomycete”. The final analysis of the ITS region consisted of 512 clearly aligned sites. Maximum likelihood analysis resulted in one ML tree with final ML optimization Likelihood: -3018.425987. Using a phylogenetic species criterion in combination with the Automatic Barcode Gap Discovery (ABGD) method, a total of 32 species-level clades were circumscribed in the ITS gene tree, with 21 species-level clades in *Cudonia* and 11 in *Spathularia*.

Maximum likelihood analysis of the nrLSU data yielded an optimal ML tree with a log likelihood score -4099.381378 (Figure S1). The nrLSU phylogeny recovered a strongly supported Cudoniaceae clade derived from within the Rhytismataceae. Within Cudoniaceae clade, several subclades within *Cudonia* and *Spathularia* were recovered but bootstrap support for the nrLSU dataset was generally weak compared with the ITS dataset.

The optimal ML tree based on the *rpb2* dataset had a log likelihood score of -3549.890566 (Figure S2). The *rpb2* analysis recovered a monophyletic Cudoniaceae clade and a monophyletic *Cudonia* with strong bootstrap support. *Spathularia* was paraphyletic and divided into two major clades. One *Spathularia* clade, represented by *S. velutipes* Cooke & Farl. and allies, is resolved as sister to *Cudonia*. The other *Spathularia* clade, represented by *S. flavida* and two undescribed species, is sister to the rest of the group.

The *tef-1α* ML analysis produced a tree with the optimization likelihood value -2044.088513 (Figure S3). The *tef-1α* analysis recovered a strongly supported, monophyletic Cudoniaceae and genus *Spathularia,* but *Cudonia* was paraphyletic with *Spathularia* nested inside of *Cudonia*.

### Phylogenetic species delimitation

**Figure 2:**
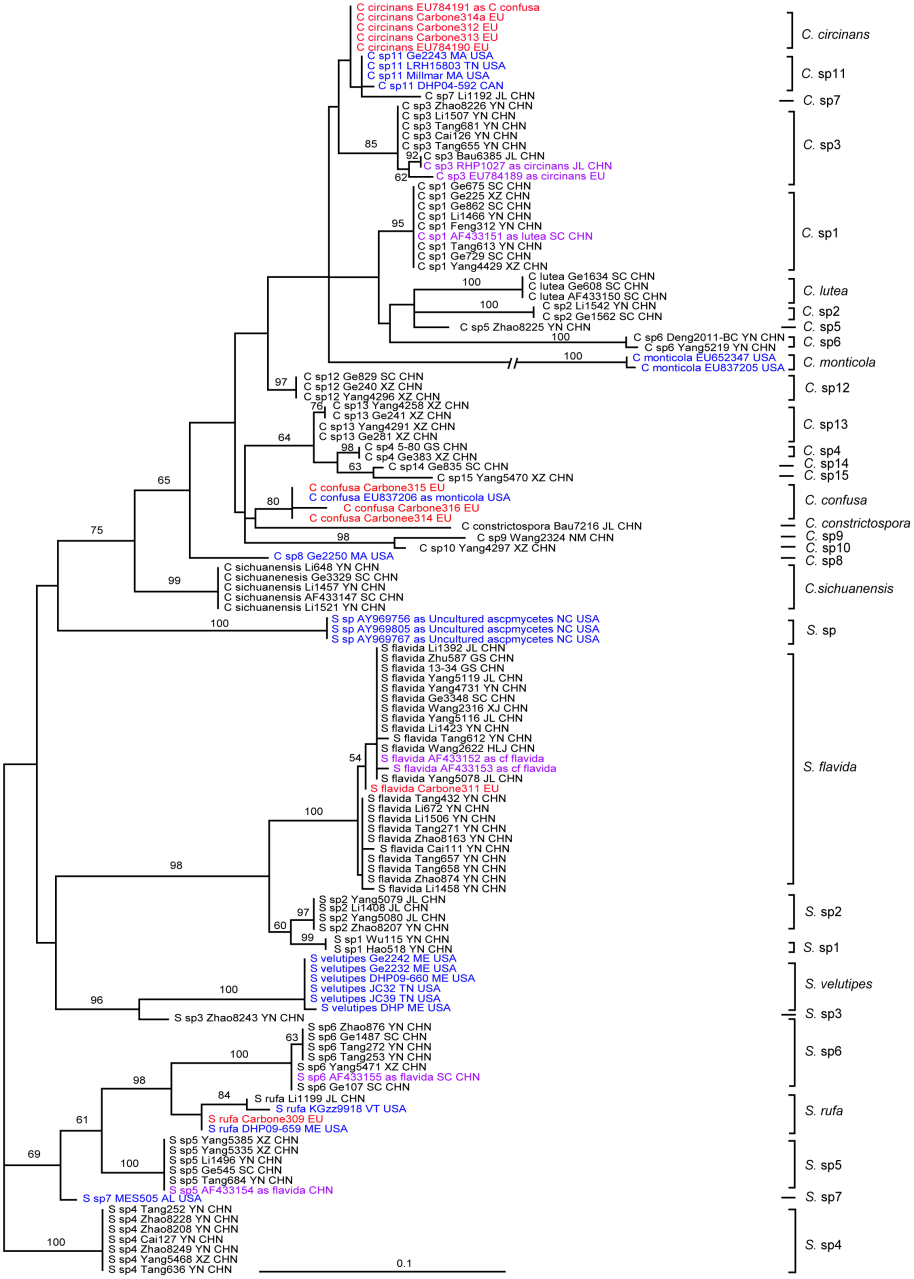
Maximum likelihood phylogeny of *Cudonia* and *Spathularia* species based on 127 ITS ribosomal DNA sequences. ML bootstrap support values are shown above the internal branches. Sequences from Asia are in black text, sequences from North America are in blue text, and sequences from Europe are in red text. Mislabeled sequences from GenBank and sequences from misidentified herbarium collections are shown in purple text.

In total, 32 putative species of *Cudonia* and *Spathularia* were recognized based on ITS data using the ABGD method (Figure 2), with 21 species-level clades in *Cudonia* and 11 in *Spathularia*. Among the 32 putative species, 20 species (8 in *Spathularia* and 12 in *Cudonia*) were further confirmed by the multilocus genealogical phylogenetic species recognition approach since there are multiple collections and sequences from multiple loci available for these species; two additional species (*Cudonia monticola* and *Spathularia* sp.) were recognized as distinct species because they form monophyletic clades with strong statistical support in the ITS analysis (both got 100 bootstrap support).

Ten putative species represented by only a single specimen were identified based on the uniqueness of their ITS sequences. The ITS variation within species ranged from 0 to 0.005 in the known *Cudonia* and *Spathularia* species (Table S4). *Cudonia monticola* displayed the highest ITS rDNA hetereogeneity (0.005), and was chosen as the cutoff value for the species identification using ITS sequences. Taking this value as a threshold, *Cudonia* ‘sp5’, *Cudonia* ‘sp7’, *Cudonia* ‘sp8’, *Cudonia* ‘sp9’, *Cudonia* ‘sp10’, *Cudonia* ‘sp14’, *Cudonia* ‘sp15’, *Spathularia* ‘sp3’ and *Spathularia* ‘sp7’ were recognized as putative species based on the ABGD analyses (Table S4).

### Phylogenetic analysis of *Cudonia* and *Spathularia* based on a multi-gene dataset

Using the reciprocal 70% bootstrap support approach, there is very little evidence for incongruence among the individual genes. Accordingly, all four genes were concatenated for ML and BI analyses to assess the phylogeny of *Cudonia* and *Spathularia*.

**Figure 3:**
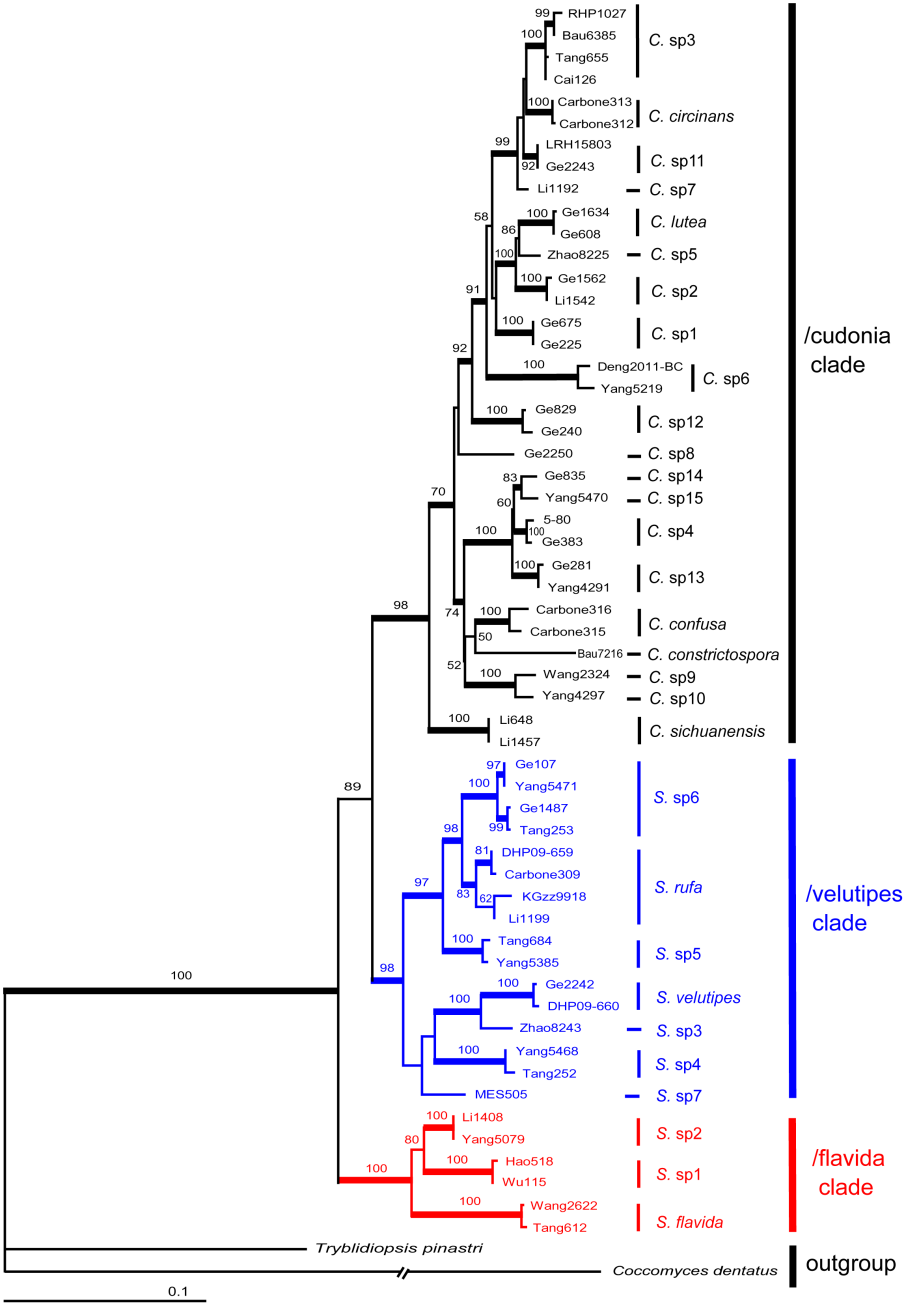
Four-locus (ITS, nrLSU, *rpb2* and *tef-1α*) maximum likelihood (ML) phylogeny depicting phylogenetic relationships among 58 sequences of *Cudonia* and *Spathularia*. Bootstrap support values from ML (1000 bootstrap replicates) analyses are shown above the internal branches. Bayesian posterior probabilities (BPP) greater than 0.95 are indicated by thickened branches. Branch lengths are proportional to the average number of substitutions per site except for dashed lines, which indicate longer branches that are not shown to scale.

*Cudonia monticola* and the novel “uncultured ascomycete” from North Carolina forest soil were not included in the multilocus phylogenetic analyses because only ITS sequences were available for these taxa and these sequences were challenging to align with other members of *Cudonia* and *Spathularia*. Because the sampling is unbalanced for certain taxa in the 96 specimen phylogeny (for instance, *Spathularia flavida* has 15 collections in Figure S5), and to display the phylogeny effectively, we randomly chose two collections with the most complete sequences for each species and built a 58 specimen phylogeny for *Cudonia* and *Spathularia* (Figure 3). This analysis utilized 2,583 aligned nucleotides from 58 specimens, including representatives of all major clades. After excluding ambiguously aligned positions (mainly gaps introduced by the outgroup taxa), the final alignment was 2,458 nucleotides in length. The likelihood value of the final ML tree was lnL =  -10065.428556 (Figure 3), and the likelihood value of the consensus Bayesian tree were lnL  =  -9973.42 and -9979.74 for the cold chain runs.

The topology of the tree identified by ML analysis (see Figure 3) was almost identical to that found by the Bayesian analyses. Regardless of the analytical method, a highly resolved phylogeny of *Cudonia* and *Spathularia* was recovered from the multi-gene dataset. The family Cudoniaceae and the genus *Cudonia* were both strongly supported as monophyletic groups (Figure 3) whereas *Spathularia* was divided into two major clades: the /flavida clade and /velutipes clade. These three major clades (e.g. /cudonia, /flavida, and /velutipes) were recovered with strong support by both methods.

The /flavida clade forms a sister clade to a clade jointly formed by /velutipes clade and /cudonia clade. The /velutipes and /cudonia clades are supported as sister to one another, but this relationship received moderate to weak support (ML = 88, BI = 0.90). Overall, the multi-gene analysis identified 30 species level clades and different methods generally recovered similar relationships between the groups. The only difference is that *S.* sp7 is sister to a clade formed by *S.* sp5, *S.* sp6 and *S. rufa* in the Bayesian analyses, instead of sister to a clade formed by *S.* Sp3, *S.* Sp4, and *S. velutipes* in the ML analysis. Within *Cudonia* we identified 20 putative species, with *C. sichuanensis* resolved as the earliest diverging clade that is sister to the remaining taxa with strong statistical support (Figure 3). Among these 20 putative species, scientific names can only be confidently applied to five species whereas the other 15 clades appear to represent novel taxa due to their unique morphology and/or consistent and identifiable habitat preferences. Similarly, of the 10 *Spathularia* species-level clades that were recovered from the phylogeny, names can only confidently be applied to three clades. The /flavida clade is comprised of three taxa; the widely distributed *Spathularia flavida* Pers. and two undescribed species, *Spathularia* sp1 and sp2. *Spathularia* sp1 from Southwestern China and *Spathularia* sp2 from Northeastern China are sister taxa that form a joint clade that is sister to *S. flavida.* The /velutipes clade includes *Spathularia velutipes* Cooke & Farl., *Spathularia rufa* Schmidel and five unidentified species from southwestern China and/or North America.

### Divergence time estimation and ancestral area reconstruction for *Cudonia* and *Spathularia*

**Figure 4:**
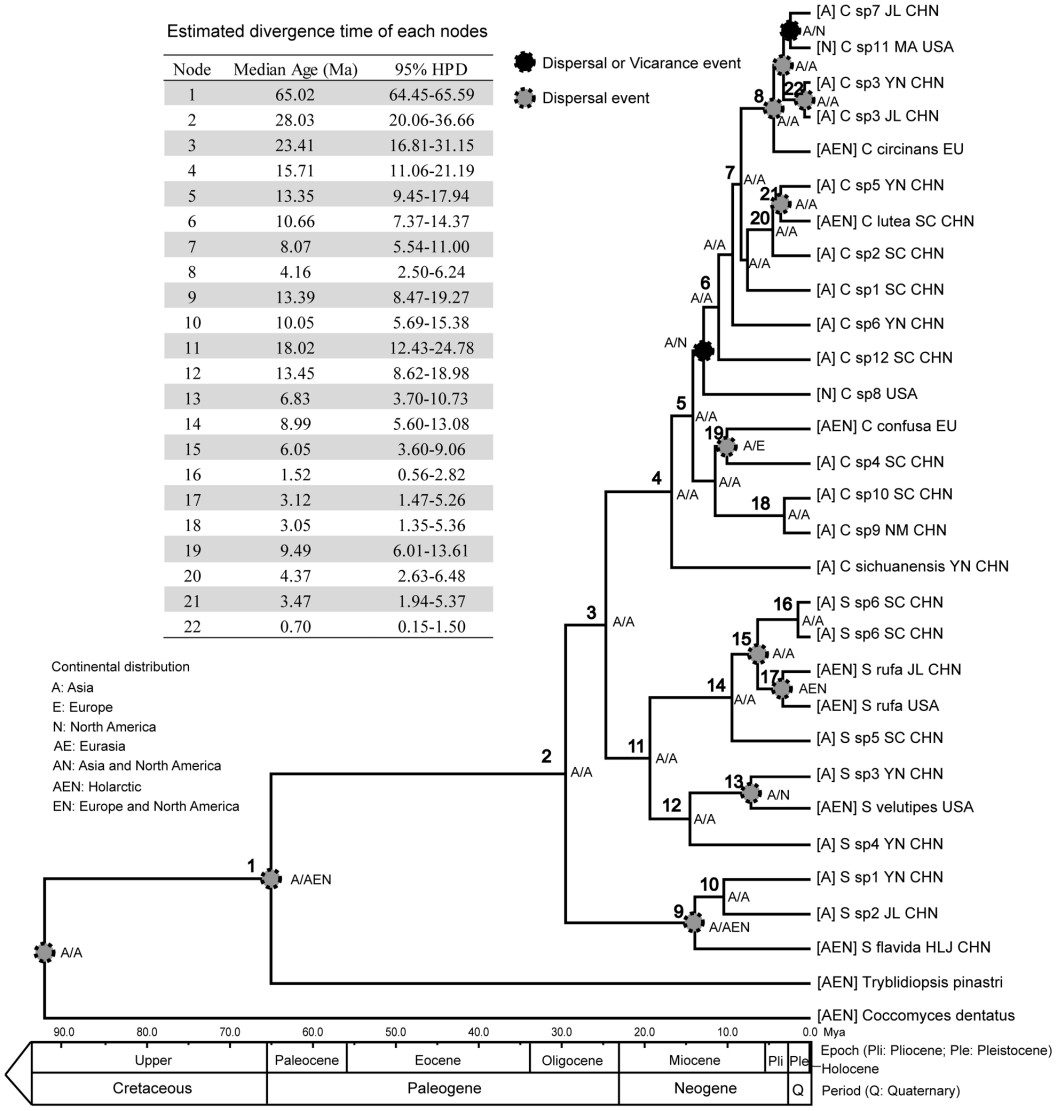
Ancestral area reconstruction and chronogram of clade of *Cudonia* and *Spathularia* based on BEAST analyses using *Cudonia* stem (65.03 ± 0.29 MYA, node 1) as calibration. Estimated mean divergence times and 95% highest posterior density of resolved nodes in phylogenetic analyses are summarized in the upper left panel. Geological time scale according to Gradstein et al. (2004) is shown along the bottom for scale.

The estimated mean divergence time of Cudoniaceae from *Tyblidiopsis pinastri* (Rhytismatales) was during the beginning of the Paleogene in the Early Tertiary at 65.03 ± 0.29 Mya (95% highest posterior density [HPD] interval: 41.97-90.21). Our analysis suggests that diversification within *Cudonia* and *Spathularia* began during the Middle Oligocene 28.65 ± 0.14 Mya (95% HPD: 15.25-42.87) in late Paleogene (Figure S4). Divergence time of the Cudoniaceae members estimated using the above values (Cudoniaceae stem and crown) as calibration points are summarized in Figure 4.

From the chronogram in Figure 4, */*flavida clade is estimated to have diverged from /velutipes clade and /cudonia clade at 28.03 Mya (95% HPD: 20.06-36.66) in the Oligocene. The divergence between /velutipes clade and /cudonia clade is estimated to have occurred between late Oligocene and early Miocene (23.41 mya, with 95% HPD 16.81-31.15). The /velutipes clade is estimated to have diverged at 18.02 Mya (12.43-24.78) in the early Miocene, and the /cudonia clade is estimated to have diverged at 15.71 mya (95% HPD: 11.06-21.19) in the Middle Miocene. Subsequent divergence times within the /velutipes clade were estimated for the /velutipes subclade at 13.45 Mya (95% HPD: 8.62-18.98) and for the /rufa subclade at 8.99 Mya (95% HPD: 5.60-13.08). Speciation events within the /rufa subclade were estimated to have occurred during the Pliocene and Pleistocene. *Spathularia* sp3 diverged from /velutipes in the late Miocene.

Speciation events of most /cudonia clades were estimated to be diverged in Miocene and Pliocene, with a few diverged within Pleistocene. The morphologically unique species *Cudonia* sp9 and *Cudonia* sp10 were estimated to have diverged from *C. confusa* and *Cudonia* sp4 at approximately 9.49 Mya (95% HPD: 6.01-13.61), and they split from each other at roughly 3.05 Mya (95% HPD: 1.35-5.36).

Based on the dispersal-extinction-cladogenesis analysis generated with the LAGRANGE software package, our ML-based ancestral area reconstruction analysis determined that the most recent common ancestor of Cudoniaceae most likely lived in Asia (Figure 4), followed by dispersal of the /flavida clade to Europe and North America. Similarly, we also inferred that the most recent common ancestor of both the /velutipes clade and /cudonia clade is likely to have arisen in Asia.

## Discussion

### Molecular phylogeny and species diversity of *Cudonia* and *Spathularia*

A recent study estimated that there are 5.1 million species of fungi on Earth but only about 100,000 fungi are currently described in the literature [3]. Two important reasons for this disconnect between estimated and described fungal species are: 1) there has been inadequate sampling of various fungal groups in many world regions, and 2) many reproductively isolated, cryptic fungal species are “hidden” within morphological species complexes and can only be elucidated by in-depth molecular studies.

Our results suggest that *Cudonia* and *Spathularia* are two such understudied genera that have been poorly sampled and contain cryptic species diversity within complexes of morphologically similar taxa. *Cudonia* and *Spathularia* have been under-collected, overlooked, and rarely studied using molecular tools. In this study, we use sequences of ITS, nrLSU, *rpb2* and *tef-1α* to elucidate diversity within *Cudonia* and *Spathularia* and also to clarify broader biogeographical and evolutionary patterns within the group. Our results support the hypothesis of a monophyletic Cudoniaceae, as suggested by previous molecular studies that included only a few *Cudonia* and *Spathularia* species [27]-[32].

Our molecular analysis of *Cudonia* and *Spathularia* has several implications for the taxonomy and systematics of this group. First, our individual (Figure S1) and multi-gene analyses (Figure S4) agree with the phylogenetic results of Lantz et al. [27] that the Cudoniaceae is nested within the Rhytismataceae. Since this situation renders Rhytismataceae a paraphyletic group, we suggest that Cudoniaceae be abandoned in favor of a broadly circumscribed Rhytismataceae.

A second major issue is that *Cudonia* is a monophyletic group whereas *Spathularia* is not. At first glance it might seem appealing to split the genus *Spathularia* in two since there are already two generic names available: the /flavida clade corresponds to *Spathularia*, and the /velutipes clade to *Spathulariopsis* Maas Geest. Geesteranus erected *Spathulariopsis* to accommodate *Spathularia velutipes* based on hemiangiocarpous development (the hymenium becomes exposed before maturity) and the hyphal structure of the medulla layer in the stipe [68]. However, similar structures can also be found in *Cudonia sichuanensis* [9], *C. lutea* [14], *Cudonia* ‘sp12’ (Z-W Ge and ZL Yang, personal observation), and early developmental stages of other *Cudonia* and *Spathularia* species [13], [15], [16]. Imai also erected a separate genus, *Pachycudonia* S. Imai, for *Cudonia constrictospora* S. Ito et S. Imai [69]. He suggested that the long-tailed asci, elongated spores with constricted middle sections, and paraphyses with coiled ends were diagnostic for this genus [69]. However, our phylogenetic placement of *C. constrictospora* and *C. monticola* in the /cudonia clade suggests that *Pachycudonia* can be considered a synonym of *Cudonia.*

Instead of recognizing three genera we suggest a more straightforward approach of recognizing the single genus *Spathularia*. This genus name has priority because it was described by Persoon in 1794 whereas *Cudonia* was not described until 1849 [70], [71]. The main rationale for recognizing the single genus *Spathularia* is that there are very few diagnostic morphological characters to readily separate the major clades in this group of fungi. Traditionally, the main character that has been used to distinguish *Spathularia* from *Cudonia* was that *Spathularia* species have flattened, spatula-shaped ascoma (e.g. *S. flavida* and *S. velutipes* in Figure 1) whereas *Cudonia* species have rounded ascoma (e.g. *C. circinans, C. lutea, C. confusa* in Figure 1). However, we have documented two apparently undescribed species from China that are morphologically unique and are not easily pigeonholed into *Cudonia* or *Spathularia*. *Cudonia* ‘sp10’ has ascoma that are intermediate between the spatulate and rounded forms whereas *Cudonia* ‘sp9’ forms smooth, clavate ascoma that macroscopically resemble the fruiting bodies of the basidiomycete genus *Clavariadelphus* (Figure 1). Since major nomenclatural amendments are beyond the scope of this paper, we retained the genus names *Cudonia* and *Spathularia* to describe the overall morphology of our undescribed species in this publication.

In addition to clarifying the major groups within *Spathularia* and *Cudonia,* and assessing their relationships to one another, our molecular analyses also identified 32 distinct species-level clades based on our herbarium collections and GenBank sequences (30 species were identified based on both a multi-locus phylogenetic species recognition approach and the Automatic Barcode Gap Discovery (ABGD) method whereas the remaining two species were identified based only on ITS sequences using the ABGD approach[62]). We detected high species diversity in East Asia, including 16 species-level clades in *Cudonia,* and 8 in *Spathularia*. The large number of species in East Asia suggests that this region is a center of diversity for *Spathularia* and *Cudonia*. However, since our field sampling was most intense in East Asia, it is also possible that other world regions host unrecognized cryptic diversity of *Spathularia* and *Cudonia*. Our analysis includes 6 out of 13 named species of *Cudonia* and 3 out of 6 named *Spathularia* species. There are currently five described *Cudonia* species (*C. circinans, C. lutea*, *C. grisea*, *C. monticola*, and *C. ochroleuca*) and three described *Spathularia* species (*S. clavata S. rugosa* and *S. velutipes*) recognized in North America [14], [16], [21], [72]. We identified two additional North American species based on herbarium specimens (*Cudonia* ‘sp8’ and *Spathularia* ‘sp7’) as well as a clade that is only known from soil DNA sequences (*Spathularia* ‘sp’). In Europe, two *Cudonia* species (*C. circinans, C. confusa*), and two *Spathularia* species (*S. flavida* and *S. rufa*) are commonly recognized [19], [20]. In addition, several infraspecific varieties of *S. flavida* have been described in Europe [73], [74], but were subsequently ignored as synonyms by most mycologists. Based on the unexpectedly high diversity and the large number of “hidden” taxa detected in this study, we expect that these varieties probably represent distinct species and will need to be reevaluated with molecular techniques.

Our molecular phylogeny points to the need for a major taxonomic revision within *Cudonia* and *Spathularia*. In addition to the major taxonomic changes highlighted above, it will be important to identify and describe the putative new species that we detected in East Asia and also to reevaluate the previously described species in Europe and North America based on molecular data. Results from the ITS phylogeny suggest that specimens in *Cudonia* and *Spathularia* are routinely mislabeled. For example, sequence EU837206 was deposited as *Cudonia monticola* but is resolved in the *C. confusa* clade, sequence EU784191 was deposited as *C. confusa* but falls in the *C. circinans* clade, and sequence EU784189 was deposited as *C. circinans* but is in fact *Cudonia* ‘sp3’. In *Spathularia*, two sequences deposited as *S. flavida* (AF433154, AF433155) are resolved in the *Spathularia* ‘sp6’ clade and *Spathularia* ‘sp5’ clade, respectively. Three soil clone sequences from North Carolina (USA) pine forests (as “Uncultured Ascomycete” – AY969756, AY969805, AY969767) apparently represent a novel *Spathularia* species. Further taxonomic work will also help to clarify the species delimitations of described taxa and may help to alleviate the large number of misidentified sequences deposited in GenBank (Figure 2).

Based on our extensive field collections (Table S1), we have observed that some species have consistent and identifiable habitat preferences that have been mostly ignored in previous studies. For example, *Spathularia velutipes* is usually found on decayed logs, *Spathularia* sp4 usually fruits directly on soil in angiosperm-dominated forests, and *Cudonia sichuanensis, Cudonia* ‘sp10’ (Figure 1E), and *Cudonia* ‘sp12’ (Figure 1G) all fruit among mosses in dense, mature coniferous forests. These observations suggest that ecological factors may be useful in clarifying the taxonomy and ecology of species in this group.

### Morphological evolution and adaptations to a temperate environment

Most species within Rhytismatales produce their spores within a dark stroma that is immersed in host tissues and is sometimes covered by a layer of fungal hyphae [17]. The vast majority of species in the order also produce fruiting bodies that are small, thin, and inconspicuous whereas those of *Spathularia* and *Cudonia* are relatively noticeable and large (stipes up to 7 cm long and pilei up to 2 cm broad). Like most other Rhytismatales, *Spathularia* and *Cudonia* species also have a veil that shields the hymenium during development (Figure 1G. *Cudonia* ‘sp12’). This type of development (called “hemiangiocarpous development”) may help to protect the hymenium from adverse environmental conditions such as freezing or desiccation. We also detected the production of conidia (asexual spores) directly on or within the fruiting bodies of *Cudonia* ‘sp10’ and ‘sp12’, and this phenomenon was previously reported for *C. lutea, C. circinans, S. flavida* and *S. velutipes* [15], [16]. We think this strategy of asexual spore production may be an additional adaptation to environmental stress, but more studies are needed to fully understand the role of conidia in the biology of *Spathularia* and *Cudonia*.

Members of the order Rhytismatales are considered plant endophytes or pathogens [10]-[12], [17], whereas *Spathularia* and *Cudonia* have been considered by some authors to be white-rot saprobes [75]. In an evolutionary context, the transition from an endophytic or pathogenic to a saprobic lifestyle may have been an important evolutionary event during the history of *Spathularia* and *Cudonia*. It is possible that the large ascoma are an adaptation to saprotrophism; it has been hypothesized that large fruiting bodies can generate more spores and their fleshy stalks can more easily rise above the surface of the leaf litter. The idea that fleshy sporocarps are more likely to occur in saprobic taxa has also been suggested as a wider pattern within Ascomycota [17]. Given that the ecology of these fungi is so poorly known, it also remains possible that *Spathularia* and *Cudonia* species have cryptic plant endophytic, mycoparasitic, or plant pathogenic stages. We think this is unlikely, however, because most environmental sequences of *Spathularia* and *Cudonia* are from soil. For example, we detected three sequences from organic soil in a North Carolina pine forest (AY969756, AY969805 and AY969767), one sequence from soil under *Pinus sylvestris* in Sweden (FJ475669), and one from soil in a mixed forest in New Hampshire (HQ022046). In contrast, we detected only one environmental DNA sequence from roots; sequence HQ260170 was recovered from *Ericaceae* roots in Alaska. These environmental sequence data suggest that *Spathularia* and *Cudonia* are probably most common in soil but also reinforce that taxa in this group tend to be rare and their ecology is not well understood.

### Biogeographic history of *Cudonia* and *Spathularia* and their diversification in East Asia

The divergence patterns of fungi are still poorly understood because there are only a handful of fossils that can be used to definitively date the emergence of particular fungal clades [76], [77]. However, molecular dating and phylogenetics tools have proved useful for estimating the divergence times and centers of origin for some fungal groups. For example, recent studies have examined patterns and timing of evolution in ascomycetes such as Hypocreales [78], *Morchella* [41], [79], *Tuber* [80], [81], *Golovinomyces* [82], and some lichen-forming clades [83]-[85].

Our relaxed clock analyses, which allow the molecular rate to vary among clades, suggest the diversification of Cudoniaceae started around 28.65 Mya (95% HPD: 42.87-15.25) (Node 2 in Figure 4). Compared to some other Pezizomycetes that are thought to have diverged during the Cretaceous (e.g. *Morchella* [41]; *Tuber* [80], our analysis suggests that Cudoniaceae is a relatively young clade whose divergence times are more similar to lichen-forming fungi in Parmeliaceae (Lecanoromycetes) [83].

Our ancestral area reconstruction analyses suggest that Cudoniaceae may have evolved in Asia and then later dispersed to Europe and North America (Figure 4). We detected 24 species of *Spathularia* (8 species) and *Cudonia* (16 species) in Asia whereas only 9 species were found in North America and only 5 species were found in Europe. Some of this high species diversity in Asia versus other continents may reflect our intensive field sampling in Asia and the fact that some uncommon species originally described from North America (e.g. *Cudonia ochroleuca* [Cooke & Harkn.] E.J. Durand, *Cudonia grisea* Mains) or Europe (*Spathularia alpestris* [Rehm] Rahm, *Spathularia crispata* Fuckel) were not available for molecular analysis. However, Asian *Spathularia* and *Cudonia* species exhibit higher morphological diversity than species from other regions (including ascocarp shape and color, spore morphology, stipe anatomy, and the structure of the hymenial veil). These fungi are also found in a wide variety of habitats in Asia, including alpine, subalpine, and subtropical habitats. Furthermore, Asia has been implicated as a center of diversity for several major groups of fungi such as *Amanita*, *Boletus*, *Flammulina*, and *Morchella* [37], [79], [86], [87], [106].

The dates for major radiations in *Spathularia* and *Cudonia* are estimated to coincide with one of the two pulses of rapid uplifting of the Tibetan plateau which occurred during 25-30 Mya [88]. This time period was characterized by a global long-term glaciation and massive geological uplifting in the Himalaya region in Asia that contributed to both regional and global cooling trends [89]-[94]. During this time, the mountains of northwestern China became arid and subtropical evergreen forests were restricted to southern and coastal Asia [95]. These large-scale geological and climatic events in Oligocene (e.g. the period of global cooling from 34–23 Mya and the uplift of the Tibetan plateau) are strongly correlated with diversification in *Spathularia* and *Cudonia*. This unique geological history of the Tibetan Plateau is thought to have generated heterogeneity in the soils, climate, and elevation across the region [87]. These changes are implicated as potential drivers of evolutionary radiations in many organisms, including key clades of forest trees [96], [97]. The rapid drop in temperature during this period may have stimulated the radiation of other cold-adapted groups in fungi such as *Amanita* and *Boletus* [37], [38], in plants such as *Quercus* [98], [99], and in insects such as *Bombus* and *Myrmica* [100], [101]. We hypothesize that the global cooling and expansion of temperate forests across south Asia may have triggered evolutionary radiations in this group of fungi, either directly through the separation of fungal populations into refugia or indirectly through changes in the composition of forest communities.

Long-distance spore dispersal and ancient vicariance are the main mechanisms used to explain the intercontinental distributions for fungi, although more recent movements have sometimes been due to human-mediated introductions [102]-[104]. In combination with the divergence time estimates, our LAGRANGE analyses suggest that dispersal is the dominant mechanism behind the continental distribution of *Spathularia* and *Cudonia* species. Due to the large gelatinous spore sheaths that cover ascospores at maturity (Figure 1O), we suspect that *Spathularia* and *Cudonia* ascospores are not easily wind dispersed. Rain-splash dispersal and/or insect vectors may be involved in ascospore dispersal and we hypothesize that the asexual conidia we observed might also be important for occasional, long-distance dispersal. However, more detailed ecological studies are needed. In addition to the inferred instances of dispersal, two vicariance events may also have occurred in the evolutionary history of the /cudonia clade (inferred at around 12 Mya and 3 Mya respectively – Figure 4). However, these events can also be explained by dispersal because the Bering Land Bridge has been a viable route for terrestrial plants (and fungi) since the Paleocene [105]. One species within the /cudonia clade, *Cudonia monticola*, demonstrated an extremely long branch within the ITS phylogeny (Figure 2). This may be a signature of rapid evolution and/or a selection bottleneck during dispersal to western North America but more work is needed to resolve the placement and history of this species.

This study clearly shows that the species-level diversity of *Spathularia* and *Cudonia* is higher than was previously thought and we have increased the total number of putative taxa to 32 phylogenetic species based on a combination of multi-gene phylogenetic analyses and the Automatic Barcode Gap Discovery (ABGD) method. Although the diversity of *Spathularia* and *Cudonia* was particularly high in East Asia and our analyses suggest that this region is likely an important center of diversity for the group, the finding of many apparently new cryptic species based on molecular data suggests that other global regions may also hold unsampled or cryptic diversity. Accordingly, the biogeographic hypotheses outlined here must remain preliminary until more complete sampling is completed in key parts of Europe and North America. We suggest that additional sampling of *Spathularia* and *Cudonia* will further elucidate the systematics, ecology, and evolution of this group of fungi.

## Supporting Information

Figure S1 Maximum likelihood phylogeny of *Cudonia* and *Spathularia* species based on 113 nrLSU sequences with bootstrap values for 1000 replicates shown above the internal branches. Species of *Cudonia* and *Spathularia* are shaded in yellow, and Rhytismataceae species are shaded in green.
(TIF)

Figure S2 Maximum likelihood tree of the *Cudonia* and *Spathularia* taxa based on 78 *rpb*2 sequences with bootstrap values for 1000 replicates shown above the internal branches.
(TIF)

Figure S3 Maximum likelihood tree of the *Cudonia* and *Spathularia* taxa based on 96 *tef-1α* sequences with bootstrap values for 1000 replicates shown above the internal branches.
(TIF)

Figure S4 Chronogram of clades of *Cudonia* and *Spathularia* based on BEAST analyses. Three calibration points were used for the initial analysis based on results from Lücking et al. (2009): the stem of Ascomycota (575 ± 37.5 Mya, node A), the stem of Pezizomycotina (460 ± 30 Mya, node B), and the crown of Pezizomycotina (360 ± 20 Mya, node C). Estimated mean divergence times and 95% highest posterior densities of major nodes are summarized the upper left panel.
(TIF)

Figure S5 Maximum likelihood (ML) cladogram for 96 specimens depicting the phylogenetic relationships among *Cudonia* and *Spathularia* based on ITS-LSU-*rpb*2*-tef-1α*. Bootstrap support values above 50 (from 1000 bootstrap replicates) are shown above the branches.
(TIF)

Table S1 GenBank accession numbers and voucher information for specimens used in molecular studies.
(DOC)

Table S2 GenBank accession numbers of GenBank sequences used in this study.
(DOCX)

Table S3 GenBank accession numbers of sequences used in the divergence time estimation.
(DOCX)

Table S4 Evolutionary divergence of ITS sequence pairs within and between putative species of *Cudonia* and *Spathularia.*
(DOC)

Data S1 The 127 specimens ITS alignment with the exact nucleotides to be excluded.
(NEX)
