# Supplementary figures and images for: Multigene Molecular Phylogeny and Biogeographic Diversification of the Earth Tongue Fungi in the Genera Cudonia and Spathularia (Rhytismatales, Ascomycota)
Source: PLoS One. 2014 Aug 1;9(8):e103457. doi: 10.1371/journal.pone.0103457 (PMC4118880; doi:10.1371/journal.pone.0103457)

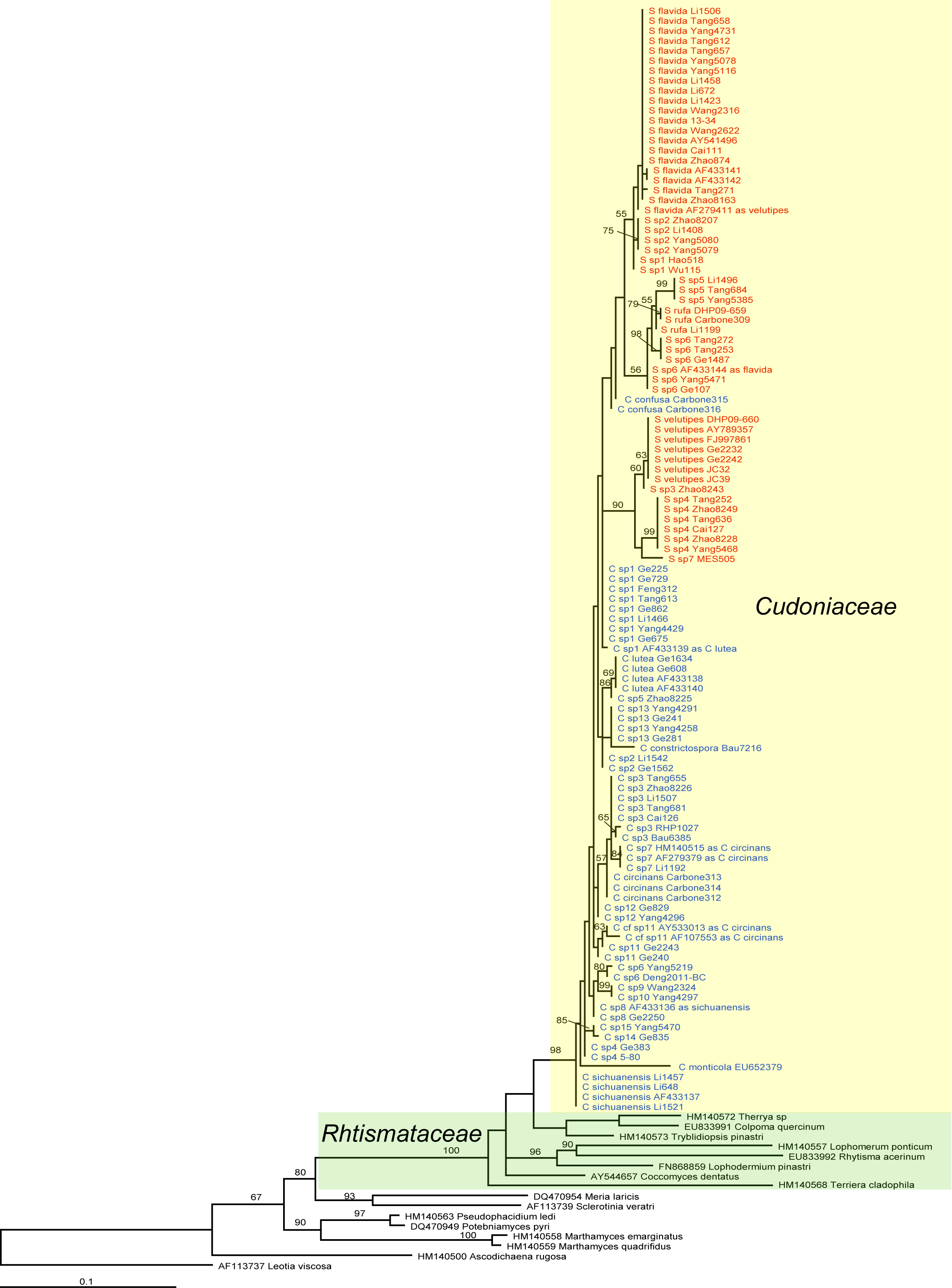

Supplement: Figure S1 — Maximum likelihood phylogeny of Cudonia and Spathularia species based on 113 nrLSU sequences with bootstrap values for 1000 replicates shown above the internal branches. Species of Cudonia and Spathularia are shaded in yellow, and Rhytismataceae species are shaded in green. (TIF) [file pone.0103457.s001.tif]

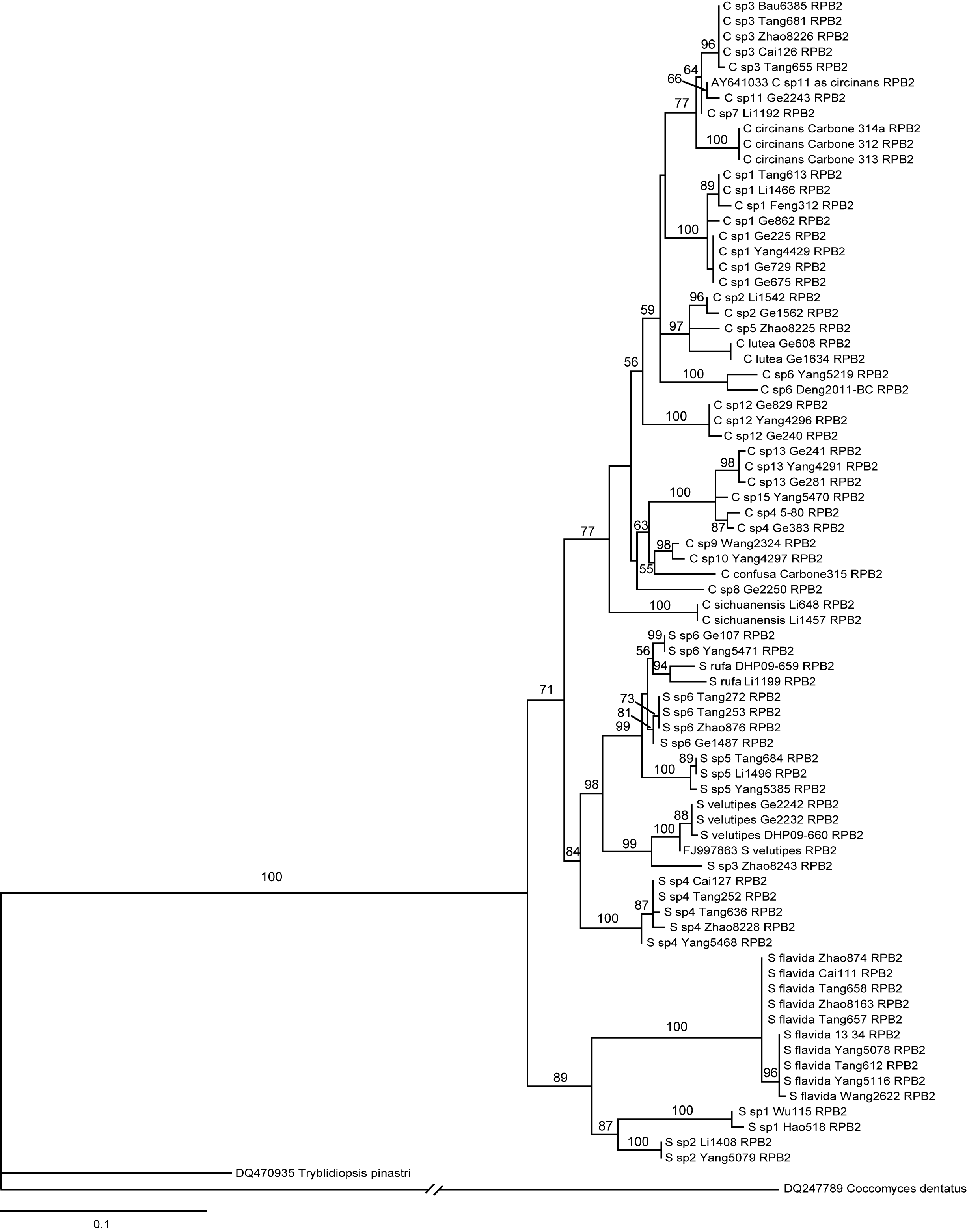

Supplement: Figure S2 — Maximum likelihood tree of the Cudonia and Spathularia taxa based on 78 rpb2 sequences with bootstrap values for 1000 replicates shown above the internal branches. (TIF) [file pone.0103457.s002.tif]

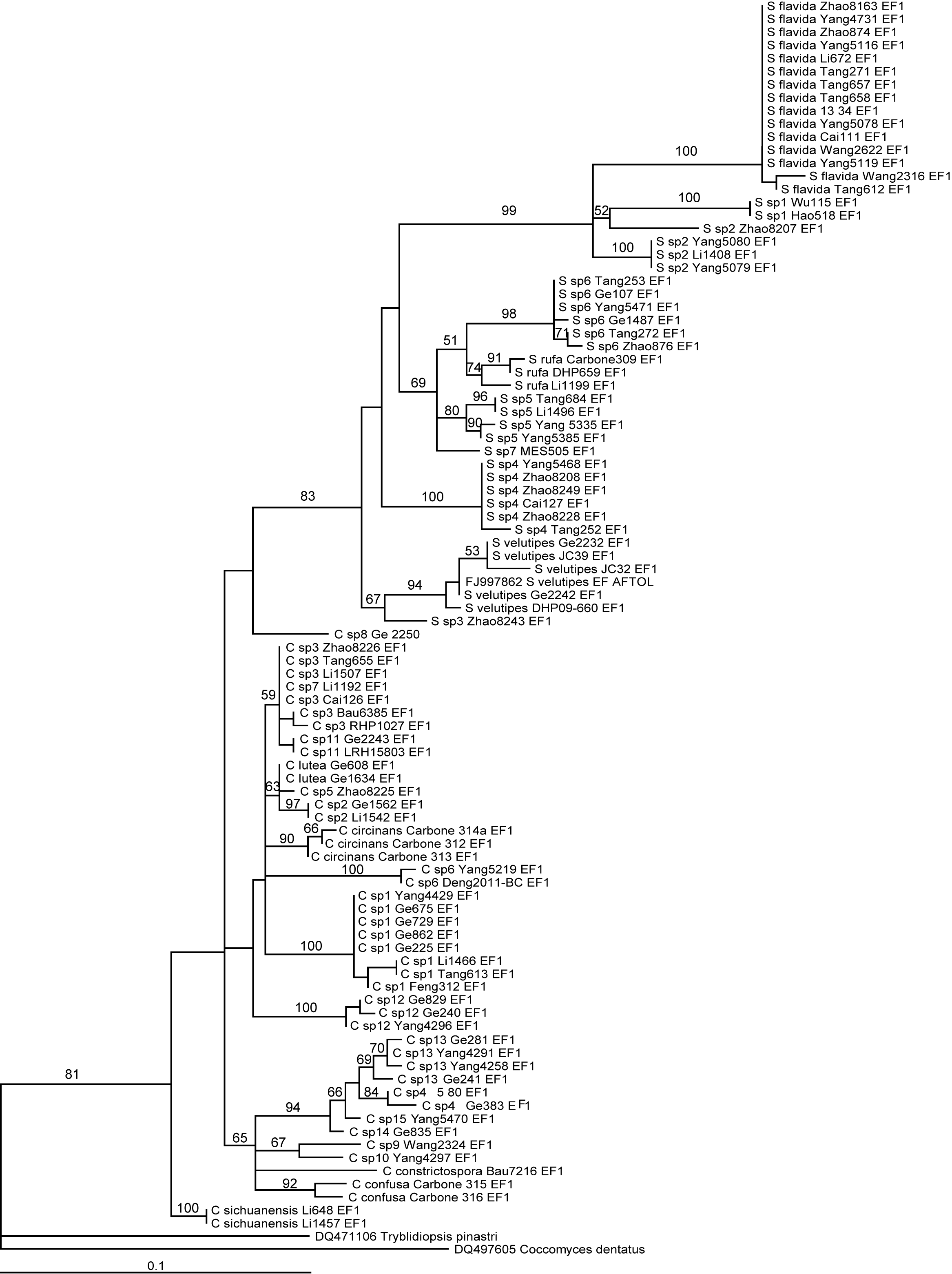

Supplement: Figure S3 — Maximum likelihood tree of the Cudonia and Spathularia taxa based on 96 tef-1α sequences with bootstrap values for 1000 replicates shown above the internal branches. (TIF) [file pone.0103457.s003.tif]

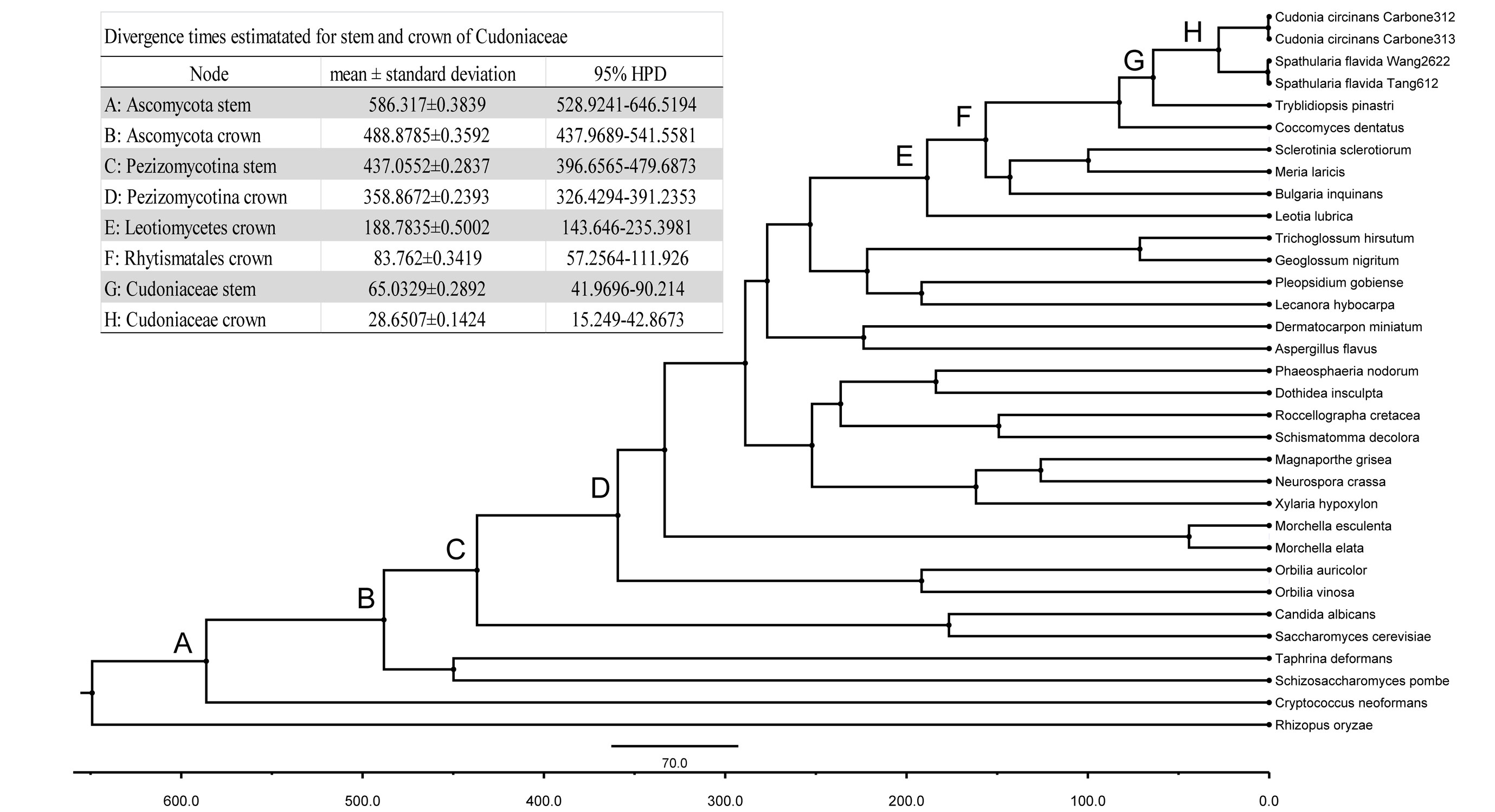

Supplement: Figure S4 — Chronogram of clades of Cudonia and Spathularia based on BEAST analyses. Three calibration points were used for the initial analysis based on results from Lücking et al. (2009): the stem of Ascomycota (575 ± 37.5 Mya, node A), the stem of Pezizomycotina (460 ± 30 Mya, node B), and the crown of Pezizomycotina (360 ± 20 Mya, node C). Estimated mean divergence times and 95% highest posterior densities of major nodes are summarized the upper left panel. (TIF) [file pone.0103457.s004.tif]

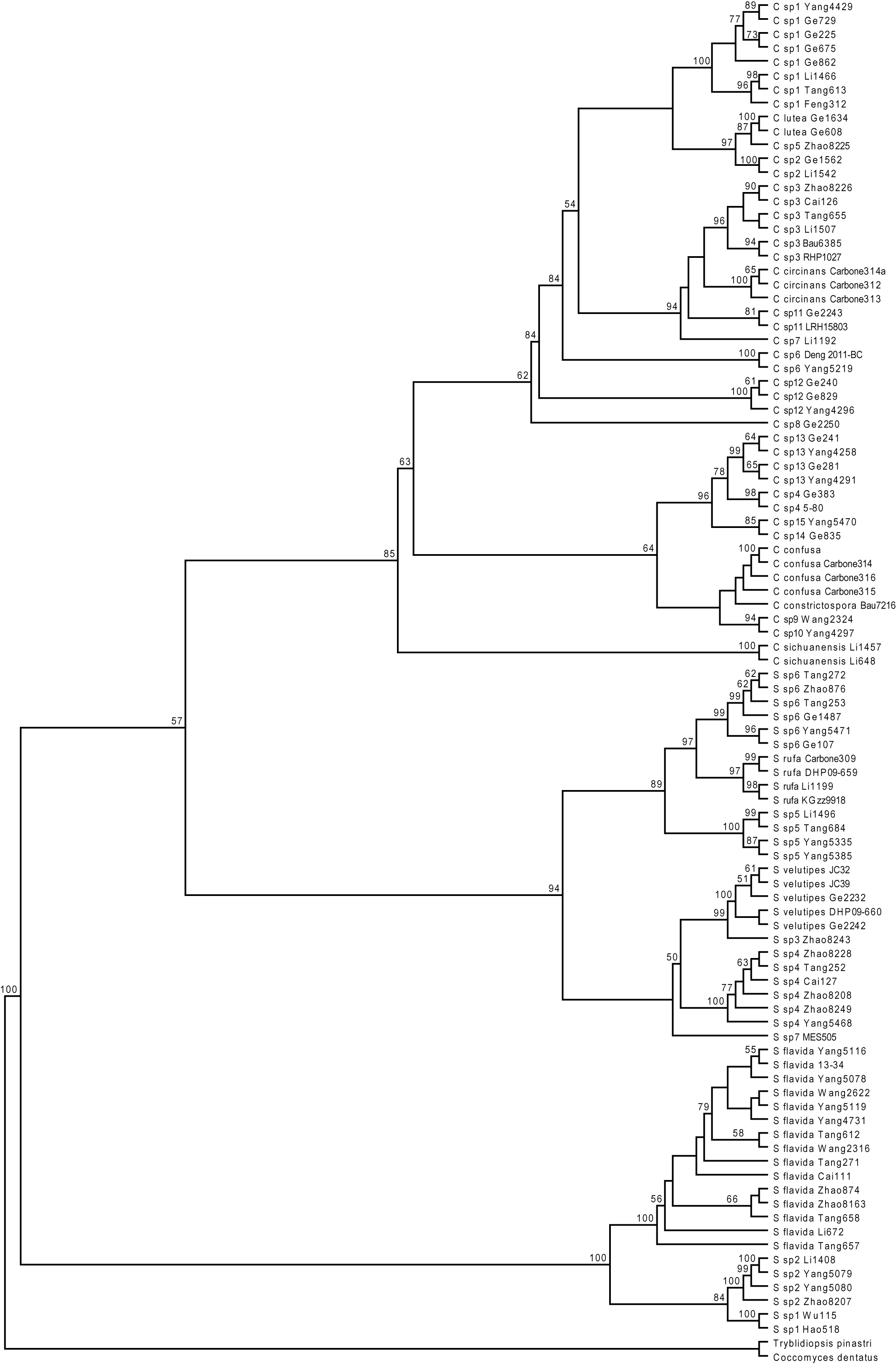

Supplement: Figure S5 — Maximum likelihood (ML) cladogram for 96 specimens depicting the phylogenetic relationships among Cudonia and Spathularia based on ITS-LSU-rpb2-tef-1α. Bootstrap support values above 50 (from 1000 bootstrap replicates) are shown above the branches. (TIF) [file pone.0103457.s005.tif]
