# Supplementary material for: Multigene Molecular Phylogeny and Biogeographic Diversification of the Earth Tongue Fungi in the Genera Cudonia and Spathularia (Rhytismatales, Ascomycota)
Source: PLoS One. 2014 Aug 1;9(8):e103457. doi: 10.1371/journal.pone.0103457 (PMC4118880; doi:10.1371/journal.pone.0103457)
Supplement: Table S1 — GenBank accession numbers and voucher information for specimens used in molecular studies. (DOC) [file pone.0103457.s006.doc]

| **Taxon id** | **collector and coll. no.** | **Coll. date** | **Organism** | **lineage** | **ITS** | **LSU** | *rpb2* | *tef-1α* | **Country** | **State or Province** |
| --- | --- | --- | --- | --- | --- | --- | --- | --- | --- | --- |
| C306 | T. Bau 6385 (HMJAU) | 2008-8-19 | *Cudonia* sp3 | /cudonia clade | KC833163 | KC833175 | KC833269 | KC833343 | China | Jilin Province (JL) |
| H674 | Y. C. Li 1507 | 2008-8-17 | *Cudonia* sp3 | /cudonia clade | KC833164 | KC833176 | ------ | KC833344 | China | Yunnan Province (YN) |
| H666 | L. P. Tang 655 | 2008-8-16 | *Cudonia* sp3 | /cudonia clade | KC833162 | KC833177 | KC833270 | KC833345 | China | Yunnan Province (YN) |
| H660 | Q. Zhao 8226 | 2008-8-18 | *Cudonia* sp3 | /cudonia clade | KC833161 | KC833178 | KC833271 | KC833346 | China | Yunnan Province (YN) |
| H654 | Q. Cai 126 | 2009-8-24 | *Cudonia* sp3 | /cudonia clade | KC833160 | KC833179 | KC833272 | KC833347 | China | Yunnan Province (YN) |
| C325 | L. P. Tang 681 | 2008-8-21 | *Cudonia* sp3 | /cudonia clade | KC833159 | KC833180 | KC833273 | ------ | China | Yunnan Province (YN) |
| H027 | DPH 04-592 (FH) | 2004-8-27 | *Cudonia* sp11 | /cudonia clade | KC833153 | ------ | ------ | ------ | Canada | Campolello Island N.B. |
| H340 | Z. W. Ge 2243 | 2009-8-14 | *Cudonia* sp11 | /cudonia clade | KC833155 | KC833181 | KC833274 | KC833348 | USA | Massachusetts (MA) |
| H037 | L. Millmar | 2009-7-17 | *Cudonia* sp11 | /cudonia clade | KC833154 | ------ | ------ | ------ | USA | Massachusetts (MA) |
| C316 | M. Carbone 312 | 2007-7-29 | *Cudonia circinans* | /cudonia clade | KC833156 | KC833182 | KC833275 | KC833349 | Switzerland | Valais, Liddes, Chandonne (EU) |
| C317 | M. Carbone 313 | 2007-8-14 | *Cudonia circinans* | /cudonia clade | KC833157 | KC833183 | KC833276 | KC833350 | Switzerland | Ticino, Olivone (EU) |
| C319 | M. Carbone 314a | 2008-8-17 | *Cudonia circinans* | /cudonia clade | KC833158 | KC833184 | KC833277 | KC833351 | Switzerland | Valais, Liddes, Chandonne (EU) |
| H670 | Y. C. Li 1192 | 2008-8-3 | *Cudonia* sp7 | /cudonia clade | KC833152 | KC833185 | KC833278 | KC833352 | China | Jilin Province (JL) |
| C280 | Z. W. Ge 608 | 2005-7-22 | *Cudonia lutea* | /cudonia clade | KC833150 | KC833186 | KC833279 | KC833354 | China | Sichuan Province (SC) |
| C283 | Z. W. Ge 1634 | 2007-7-30 | *Cudonia lutea* | /cudonia clade | KC833151 | KC833187 | KC833280 | KC833353 | China | Sichuan Province (SC) |
| C282 | Z. W. Ge 1562 | 2007-7-27 | *Cudonia* sp2 | /cudonia clade | KC833148 | KC833188 | KC833281 | KC833355 | China | Sichuan Province (SC) |
| H675 | Y. C. Li 1542 | 2008-8-21 | *Cudonia* sp2 | /cudonia clade | KC833149 | KC833189 | KC833282 | KC833356 | China | Yunnan Province (YN) |
| H658 | Q. Zhao 8225 | 2008-8-18 | *Cudonia* sp5 | /cudonia clade | KC833147 | KC833190 | KC833283 | KC833357 | China | Yunnan Province (YN) |
| C281 | Z. W. Ge 675 | 2005-7-29 | *Cudonia* sp1 | /cudonia clade | KC833146 | KC833191 | KC833284 | KC833358 | China | Sichuan Province (SC) |
| H672 | Y. C. Li 1466 | 2008-8-13 | *Cudonia* sp1 | /cudonia clade | KC833145 | KC833192 | KC833285 | KC833359 | China | Yunnan Province (YN) |
| H664 | L. P. Tang 613 | 2008-8-13 | *Cudonia* sp1 | /cudonia clade | KC833144 | KC833193 | KC833286 | KC833360 | China | Yunnan Province (YN) |
| C312 | B. Feng 312 | 2008-8-13 | *Cudonia* sp1 | /cudonia clade | KC833143 | KC833194 | KC833291 | KC833361 | China | Yunnan Province (YN) |
| C286 | Z. W. Ge 225 | 2004-7-30 | *Cudonia* sp1 | /cudonia clade | KC833139 | KC833195 | KC833287 | KC833362 | China | Xizang (Tibet) Autonomous Region (XZ) |
| C289 | Z. W. Ge 729 | 2005-8-2 | *Cudonia* sp1 | /cudonia clade | KC833140 | KC833196 | KC833288 | KC833363 | China | Sichuan Province (SC) |
| C290 | Z. W. Ge 862 | 2005-8-11 | *Cudonia* sp1 | /cudonia clade | KC833141 | KC833197 | KC833289 | KC833364 | China | Sichuan Province (SC) |
| C291 | Z. L. Yang 4429 | 2004-8-17 | *Cudonia* sp1 | /cudonia clade | KC833142 | KC833198 | KC833290 | KC833365 | China | Xizang (Tibet) Autonomous Region (XZ) |
| 1222 | X. J. Deng 2011-BC-31 | 2011-8-31 | *Cudonia* sp6 | /cudonia clade | KC833138 | KC833200 | KC833293 | KC833367 | China | Yunnan Province (YN) |
| H680 | Z. L. Yang 5219 | 2008-9-17 | *Cudonia* sp6 | /cudonia clade | KC833137 | KC833199 | KC833292 | KC833366 | China | Yunnan Province (YN) |
| C287 | Z. W. Ge 829 | 2005-8-9 | *Cudonia* sp12 | /cudonia clade | KC833134 | KC833201 | KC833294 | KC833368 | China | Sichuan Province (SC) |
| H348 | Z. L. Yang 4296 | 2004-8-4 | *Cudonia* sp12 | /cudonia clade | KC833136 | KC833203 | KC833296 | KC833369 | China | Xizang (Tibet) Autonomous Region (XZ) |
| C288 | Z. W. Ge 240 | 2004-7-31 | *Cudonia* sp12 | /cudonia clade | KC833135 | KC833202 | KC833295 | KC833370 | China | Xizang (Tibet) Autonomous Region (XZ) |
| H342 | Z. W. Ge 2250 | 2009-8-30 | *Cudonia* sp7 | /cudonia clade | KC833133 | KC833204 | KC833297 | KC833371 | USA | Massachusetts (MA) |
| H357 | Z. W. Ge 241 | 2004-7-31 | *Cudonia* sp13 | /cudonia clade | KC833131 | KC833205 | KC833325 | KC833374 | China | Xizang (Tibet) Autonomous Region (XZ) |
| H361 | Z. L. Yang 4258 | 2004-8-1 | *Cudonia* sp13 | /cudonia clade | KC833132 | KC833206 | ------ | KC833372 | China | Xizang (Tibet) Autonomous Region (XZ) |
| H362 | Z. L. Yang 4291 | 2004-8-4 | *Cudonia* sp13 | /cudonia clade | KC833130 | KC833207 | KC833321 | KC833373 | China | Xizang (Tibet) Autonomous Region (XZ) |
| C284 | Z. W. Ge 281 | 2004-8-4 | *Cudonia* sp13 | /cudonia clade | KC833129 | KC833208 | KC833323 | KC833375 | China | Xizang (Tibet) Autonomous Region (XZ) |
| C285 | Z. W. Ge 383 | 2004-8-17 | *Cudonia* sp4 | /cudonia clade | KC833127 | KC833209 | KC833324 | KC833376 | China | Xizang (Tibet) Autonomous Region (XZ) |
| H605 | 5-80 | 2010-8-27 | *Cudonia* sp4 | /cudonia clade | KC833128 | KC833210 | KC833322 | KC833377 | China | Gansu Province (GS) |
| H360 | Z. W. Ge 835 | 2005-8-9 | *Cudonia* sp14 | /cudonia clade | KC833126 | KC833211 | ------ | KC833378 | China | Sichuan Province (SC) |
| C304 | Z. L. Yang 5470 | 2009-8-4 | *Cudonia* sp15 | /cudonia clade | KC833125 | KC833212 | KC833320 | KC833379 | China | Xizang (Tibet) Autonomous Region (XZ) |
| H346 | Z. L. Yang 4297 | 2009-8-5 | *Cudonia* sp10 | /cudonia clade | KC833169 | KC833213 | KC833298 | KC833380 | China | Xizang (Tibet) Autonomous Region (XZ) |
| H371 | X. H. Wang 2324 | 2009-9-14 | *Cudonia* sp9 | /cudonia clade | KC833124 | KC833214 | KC833299 | KC833381 | China | Neimonggu Autonomous Region (NM) |
| C307 | T. Bau 7216 (HMJAU) | 2008-9-14 | *Cudonia constrictospora* | /cudonia clade | KC833168 | KC833215 | ------ | KC833382 | China | Jilin Province (JL) |
| C314 | M. Carbone 315 | 2008-8-26 | *Cudonia confusa* | /cudonia clade | KC833165 | KC833216 | KC833300 | KC833383 | Finland | Ristijarvi |
| C315 | M. Carbone 316 | 2008-8-27 | *Cudonia confusa* | /cudonia clade | KC833166 | KC833217 | ------ | KC833384 | Finland | Melahlathi |
| C318 | M. Carbone 314 | 2008-8-17 | *Cudonia confusa* | /cudonia clade | KC833167 | ------ | ------ | ------ | Switzerland | Valais, Liddes, Chandonne |
| C292 | Y. C. Li 648 | 2006-7-26 | *Cudonia sichuanensis* | /cudonia clade | KC833121 | KC833218 | KC833301 | KC833385 | China | Yunnan Province (YN) |
| C331 | Y. C. Li 1521 | 2008-8-18 | *Cudonia sichuanensis* | /cudonia clade | KC833123 | KC833219 | ------ | ------ | China | Yunnan Province (YN) |
| C328 | Y. C. Li 1457 | 2008-8-12 | *Cudonia sichuanensis* | /cudonia clade | KC833122 | KC833220 | KC833302 | KC833386 | China | Yunnan Province (YN) |
| C295 | L. P. Tang 253 | 2007-8-21 | *Spathularia* sp6 | /velutipes clade | KC833064 | KC833221 | KC833303 | KC833387 | China | Yunnan Province (YN) |
| C296 | Z. W. Ge 1487 | 2007-7-23 | *Spathularia* sp6 | /velutipes clade | KC833065 | KC833222 | KC833305 | KC833389 | China | Sichuan Province (SC) |
| C300 | L. P. Tang 272 | 2007-8-23 | *Spathularia* sp6 | /velutipes clade | KC833066 | KC833223 | KC833306 | KC833390 | China | Yunnan Province (YN) |
| C297 | Z. W. Ge 107 | 2004-7-14 | *Spathularia* sp6 | /velutipes clade | KC833068 | KC833224 | KC833307 | KC833391 | China | Sichuan Province (SC) |
| H657 | Q. Zhao 876 | 2008-7-21 | *Spathularia* sp6 | /velutipes clade | KC833067 | ------ | KC833304 | KC833388 | China | Yunnan Province (YN) |
| C305 | Z. L. Yang 5471 | 2009-8-4 | *Spathularia* sp6 | /velutipes clade | KC833069 | KC833225 | KC833308 | KC833392 | China | Xizang (Tibet) Autonomous Region (XZ) |
| H028 | Kitty Griffith ZZ9918(FH) | 1999-7-24 | *Spathularia rufa* | /velutipes clade | KC833073 | ------ | ------ | ------ | China | Norwich, Vermont (VT) |
| C313 | Y. C. Li 1199 | 2008-8-3 | *Spathularia rufa* | /velutipes clade | KC833072 | KC833226 | KC833309 | KC833393 | China | Jilin Province (JL) |
| C322 | M. Carbone 309 | 2008-8-17 | *Spathularia rufa* | /velutipes clade | KC833070 | KC833227 | ------ | KC833394 | Switzerland | Valais, Liddes, Chandonne |
| H336 | DHP 09-659 (FH) | 2009-7-17 | *Spathularia rufa* | /velutipes clade | KC833071 | KC833228 | KC833310 | KC833395 | USA | Maine, USA (ME) |
| C301 | Z. L. Yang 5335 | 2009-7-24 | *Spathularia* sp5 | /velutipes clade | KC833074 | ------ | ------ | KC833396 | China | Xizang (Tibet) Autonomous Region (XZ) |
| H673 | Y. C. Li 1496 | 2008-8-16 | *Spathularia* sp5 | /velutipes clade | KC833078 | KC833229 | KC833311 | KC833397 | China | Yunnan Province (YN) |
| H667 | L. P. Tang 657 | 2008-8-16 | *Spathularia flavida* | /flavida clade | KC833108 | KC833230 | KC833312 | KC833398 | China | Yunnan Province (YN) |
| H669 | L. P. Tang 684 | 2008-8-21 | *Spathularia* sp5 | /velutipes clade | KC833075 | KC833231 | KC833313 | KC833399 | China | Yunnan Province (YN) |
| H359 | Z. W. Ge 545 | 2005-7-18 | *Spathularia* sp5 | /velutipes clade | KC833077 | ------ | ------ | ------ | China | Sichuan Province (SC) |
| C302 | Z. L. Yang 5385 | 2099-7-29 | *Spathularia* sp5 | /velutipes clade | KC833076 | KC833232 | KC833314 | KC833400 | China | Xizang (Tibet) Autonomous Region (XZ) |
| C299 | L. P. Tang 252 | 2007-8-21 | *Spathularia* sp4 | /velutipes clade | KC833079 | KC833233 | KC833315 | KC833403 | China | Yunnan Province (YN) |
| H665 | L. P. Tang 636 | 2008-8-14 | *Spathularia* sp4 | /velutipes clade | KC833082 | KC833234 | KC833316 | ------ | China | Yunnan Province (YN) |
| H663 | Q. Zhao 8249 | 2008-8-19 | *Spathularia* sp4 | /velutipes clade | KC833081 | KC833235 | ------ | KC833401 | China | Yunnan Province (YN) |
| H661 | Q. Zhao 8228 | 2008-8-18 | *Spathularia* sp4 | /velutipes clade | KC833080 | KC833236 | KC833317 | KC833402 | China | Yunnan Province (YN) |
| H655 | Q. Cai 127 | 2009-8-24 | *Spathularia* sp4 | /velutipes clade | KC833083 | KC833237 | KC833318 | KC833404 | China | Yunnan Province (YN) |
| C311 | Q. Zhao 8208 | 2008-8-17 | *Spathularia* sp4 | /velutipes clade | KC833084 | ------ | ------ | KC833405 | China | Yunnan Province (YN) |
| C303 | Z. L. Yang 5468 | 2009-8-3 | *Spathularia* sp4 | /velutipes clade | KC833085 | KC833238 | KC833319 | KC833406 | China | Xizang (Tibet) Autonomous Region (XZ) |
| H029 | DHP (FH) | 1983-8-13 | *Spathularia velutipes* | /velutipes clade | KC833087 | ------ | ------ | ------ | USA | Orono, Maine (ME) |
| H337 | DHP 09-660 (FH) | 2009-7-17 | *Spathularia velutipes* | /velutipes clade | KC833088 | KC833239 | KC833326 | KC833407 | USA | Maine (ME) |
| H338 | Z. W. Ge 2232 | 2009-8-3 | *Spathularia velutipes* | /velutipes clade | KC833089 | KC833240 | KC833327 | KC833408 | USA | Eagle Hill, Maine (ME) |
| H339 | Z. W. Ge 2242 | 2009-8-14 | *Spathularia velutipes* | /velutipes clade | KC833086 | KC833241 | KC833328 | KC833409 | USA | Eagle Hill, Maine (ME) |
| H662 | Q. Zhao 8243 | 2008-8-19 | *Spathularia* sp3 | /velutipes clade | KC833090 | KC833242 | KC833329 | KC833410 | China | Yunnan Province (YN) |
| H883 | X. T. Zhu 587 | 2011-8-29 | *Spathularia flavida* | /flavida clade | KC833118 | ------ | ------ | ------ | China | Gansu Province (GS) |
| H676 | Z. L. Yang 5078 | 2008-8-1 | *Spathularia flavida* | /flavida clade | KC833115 | KC833243 | KC833330 | KC833411 | China | Jilin Province (JL) |
| H678 | Z. L. Yang 5116 | 2008-8-4 | *Spathularia flavida* | /flavida clade | KC833116 | KC833244 | KC833331 | KC833412 | China | Jilin Province (JL) |
| H679 | Z. L. Yang 5119 | 2008-8-5 | *Spathularia flavida* | /flavida clade | KC833117 | ------ | ------ | KC833413 | China | Jilin Province (JL) |
| H604 | 13-34 | 2009-9-7 | *Spathularia flavida* | /flavida clade | KC833114 | KC833245 | KC833333 | KC833415 | China | Gansu Province |
| H457 | X. H. Wang 2622 | 2010-8-21 | *Spathularia flavida* | /flavida clade | KC833113 | KC833246 | KC833332 | KC833414 | China | Helongjiang Province (HLJ) |
| H370 | X. H. Wang 2316 | 2009-9-9 | *Spathularia flavida* | /flavida clade | KC833112 | KC833247 | ------ | KC833416 | China | Xinjiang Uyghur Autonomous Region (XJ) |
| C326 | Y. C. Li 1392 | 2008-8-3 | *Spathularia flavida* | /flavida clade | KC833101 | ------ | ------ | ------ | China | Jilin Province (JL) |
| C327 | Y. C. Li 1423 | 2008-8-10 | *Spathularia flavida* | /flavida clade | KC833102 | KC833248 | ------ | ------ | China | Yunnan Province (YN) |
| C294 | Z. L. Yang 4731 | 2006-7-26 | *Spathularia flavida* | /flavida clade | KC833100 | KC833249 | ------ | KC833417 | China | Yunnan Province (YN) |
| C320 | M. Carbone 311 | 2008-8-17 | *Spathularia flavida* | /flavida clade | KC833106 | ------ | ------ | ------ | Switzerland | Valais, Liddes, Chandonne |
| C308 | L. P. Tang 612 | 2008-8-13 | *Spathularia flavida* | /flavida clade | KC833105 | KC833250 | KC833334 | KC833418 | China | Yunnan Province (YN) |
| H653 | Q. Cai 111 | 2009-8-22 | *Spathularia flavida* | /flavida clade | KC833107 | KC833251 | KC833335 | KC833419 | China | Yunnan Province (YN) |
| C293 | Y. C. Li 672 | 2006-7-29 | *Spathularia flavida* | /flavida clade | KC833099 | KC833252 | ------ | KC833420 | China | Yunnan Province (YN) |
| H668 | L. P. Tang 658 | 2008-8-16 | *Spathularia flavida* | /flavida clade | KC833109 | KC833253 | KC833338 | KC833422 | China | Yunnan Province (YN) |
| H659 | Q. Zhao 8163 | 2008-8-10 | *Spathularia flavida* | /flavida clade | KC833111 | KC833254 | KC833337 | KC833421 | China | Yunnan Province (YN) |
| H656 | Q. Zhao 874 | 2008-7-21 | *Spathularia flavida* | /flavida clade | KC833110 | KC833255 | KC833336 | KC833423 | China | Yunnan Province (YN) |
| C330 | Y. C. Li 1506 | 2008-8-16 | *Spathularia flavida* | /flavida clade | KC833098 | KC833256 | ------ | ------ | China | Yunnan Province (YN) |
| C329 | Y. C. Li 1458 | 2008-8-13 | *Spathularia flavida* | /flavida clade | KC833097 | KC833257 | ------ | ------ | China | Yunnan Province (YN) |
| C324 | L. P. Tang 432 | 2008-7-22 | *Spathularia flavida* | /flavida clade | KC833104 | ------ | ------ | ------ | China | Yunnan Province (YN) |
| C298 | L. P. Tang 271 | 2007-8-23 | *Spathularia flavida* | /flavida clade | KC833103 | KC833258 | ------ | KC833424 | China | Yunnan Province (YN) |
| C310 | Q. Zhao 8207 | 2008-8-17 | *Spathularia* sp2 | /flavida clade | KC833093 | KC833259 | ------ | KC833425 | China | Yunnan Province (YN) |
| H677 | Z. L. Yang 5079 | 2008-8-1 | *Spathularia* sp2 | /flavida clade | KC833094 | KC833260 | KC833340 | KC833427 | China | Jilin Province (JL) |
| H671 | Y. C. Li 1408 | 2008-8-4 | *Spathularia* sp2 | /flavida clade | KC833095 | KC833261 | KC833339 | KC833426 | China | Jilin Province (JL) |
| C323 | Z. L. Yang 5080 | 2008-8-1 | *Spathularia* sp2 | /flavida clade | KC833096 | KC833262 | ------ | KC833428 | China | Jilin Province (JL) |
| H650 | G. Wu 115 | 2009-8-22 | *Spathularia* sp1 | /flavida clade | KC833091 | KC833263 | KC833341 | KC833429 | China | Yunnan Province (YN) |
| H858 | Y. J. Hao 518 | 2011-8-14 | *Spathularia* sp1 | /flavida clade | KC833092 | KC833264 | KC833342 | KC833430 | China | Yunnan Province (YN) |
| S1 | M. E. Smith 505 | 2011-8-6 | *Spathularia* sp7 | /velutipes clade | KC833170 | KC833267 | ------ | KC833433 | USA | Fairbanks, Alaska (AL) |
| S3 | JC32 (TENN) | 2004-7-12 | *Spathularia velutipes* | /velutipes clade | KC833171 | KC833265 | ------ | KC833431 | USA | Tennessee (TN) |
| S4 | JC39 (TENN) | 2004-7-13 | *Spathularia velutipes* | /velutipes clade | KC833172 | KC833266 | ------ | KC833432 | USA | Tennessee (TN) |
| S9 | LRH15803 (TENN) | 1943-7-18 | *Cudonia* sp11 | /cudonia clade | KC833174 | ------ | ------ | KC833435 | USA | Tennessee (TN) |
| S11 | RHP1027 (TENN) | 1987-8-19 | *Cudonia* sp3 | /cudonia clade | KC833173 | KC833268 | ------ | KC833434 | China | Jilin Province (JL) |
| S66 | Z. W. Ge 3329 | 2012-8-1 | *Cudonia sichuanensis* | /cudonia clade | KC833120 | ------ | ------ | ------ | China | Sichuan Province (SC) |
| S67 | Z. W. Ge 3348 | 2012-8-2 | *Spathularia flavida* | /flavida clade | KC833119 | ------ | ------ | ------ | China | Sichuan Province (SC) |

Note: Unless the herbarium indicated in the parenthesis in column II (collector and coll. no.), voucher specimens are housed in the Cryptogamic Herbarium of the Kunming Institute of Botany, Chinese Academy of Sciences (HKAS)
