# Supplementary material for: Multigene Molecular Phylogeny and Biogeographic Diversification of the Earth Tongue Fungi in the Genera Cudonia and Spathularia (Rhytismatales, Ascomycota)
Source: PLoS One. 2014 Aug 1;9(8):e103457. doi: 10.1371/journal.pone.0103457 (PMC4118880; doi:10.1371/journal.pone.0103457)
Supplement: Table S2 — GenBank accession numbers of GenBank sequences used in this study. (DOCX) [file pone.0103457.s007.docx]

| **Table S2. GenBank accession numbers for sequences used in the phylogeny of *Cudonia* and *Spathularia*** | | | | |
| --- | --- | --- | --- | --- |
| Taxa | ITS1-5.8S-ITS2 | LSU | *rpb2* | *tef1-α* |
| *Ascodichaena rugosa* |  | HM140500 |  |  |
| *Bifusella linearis* | AY465527 |  |  |  |
| *Coccomyces dentatus* | DQ491499 | AY544657 | DQ247789 | DQ497605 |
| *Colpoma quercinum* | U92306 | EU833991 |  |  |
| *Cudonia* cf. sp11 (as *C. circinans*) |  | AF107553 |  |  |
| *Cudonia* cf. sp11 (as *C. circinans*) |  | AY533013 | AY641033 |  |
| *Cudonia circinans* | EU784190 |  |  |  |
| *Cudonia circinans* (as *C. confusa*) | EU784191 |  |  |  |
| *Cudonia confusa* (as *C. monticola*) | EU837206 |  |  |  |
| *Cudonia lutea* | AF433150 | AF433140 |  |  |
| *Cudonia lutea* |  | AF433138 |  |  |
| *Cudonia monticola* | EU652347 | EU652379 |  |  |
| *Cudonia monticola* | EU837205 |  |  |  |
| *Cudonia sichuanensis* | AF433147 | AF433137 |  |  |
| *Cudonia* sp8 (as *C. sichuanensis*) |  | AF433136 |  |  |
| *Cudonia* sp1 (as *C. lutea*) | AF433151 | AF433139 |  |  |
| *Cudonia* sp3 (as *C. circinans*) | EU784189 |  |  |  |
| *Cudonia* sp7 (as *C. circinans*) |  | AF279379 |  |  |
| *Cudonia* sp7 (as *C. circinans*) |  | HM140515 |  |  |
| *Leotia viscosa* | AY144536 | AF113737 | AY144501 |  |
| *Lophodermium pinastri* | FN868464 | FN868859 |  |  |
| *Lophomerum ponticum* |  | HM140557 |  |  |
| *Marthamyces emarginatus* |  | HM140558 |  |  |
| *Marthamyces quadrifidus* |  | HM140559 |  |  |
| *Meria laricis* | U92298 | DQ470954 | DQ470904 | DQ471073 |
| *Potebniamyces pyri* | DQ491510 | DQ470949 | DQ470900 | DQ471068 |
| *Pseudophacidium ledi* |  | HM140563 |  |  |
| *Rhytisma acerinum* | GQ253100 | EU833992 | AY641070 |  |
| *Sclerotinia veratri* | GQ377487 | AF113739 |  |  |
| *Spathularia flavida* |  | AY541496 |  |  |
| *Spathularia flavida* (as *S.* cf. *flavida*) | AF433153 | AF433142 |  |  |
| *Spathularia flavida* (as *S.* cf. *flavida*) | AF433152 | AF433141 |  |  |
| *Spathularia flavida* (as *S. velutipes*) |  | AF279411 |  |  |
| *Spathularia* sp5 (as *S. flavida*) | AF433154 |  |  |  |
| *Spathularia* sp6 (as *S. flavida*) | AF433155 | AF433144 |  |  |
| *Spathularia velutipes* |  | FJ997861 | FJ997863 | FJ997862 |
| *Spathularia velutipes* |  | AY789357 |  |  |
| *Stictis radiata* |  |  | AY641079 |  |
| *Terriera cladophila* |  | HM140568 |  |  |
| *Therrya* sp. |  | HM140572 |  |  |
| *Tryblidiopsis pinastri* | FN868463 | HM140573 | DQ470935 | DQ471106 |
| *Uncultured ascomycete* | AY969756 |  |  |  |
| *Uncultured ascomycete* | AY969805 |  |  |  |
| *Uncultured ascomycete* | AY969767 |  |  |  |
