# Supplementary material for: Multigene Molecular Phylogeny and Biogeographic Diversification of the Earth Tongue Fungi in the Genera Cudonia and Spathularia (Rhytismatales, Ascomycota)
Source: PLoS One. 2014 Aug 1;9(8):e103457. doi: 10.1371/journal.pone.0103457 (PMC4118880; doi:10.1371/journal.pone.0103457)
Supplement: Table S3 — GenBank accession numbers of sequences used in the divergence time estimation. (DOCX) [file pone.0103457.s008.docx]

| **Table S3. GenBank accession numbers for sequences used in the divergence time estimation** | | | | |
| --- | --- | --- | --- | --- |
| Taxa | 5.8S | LSU | *rpb2* | *tef1-α* |
| *Aspergillus flavus* | JN812065 | AB363745 | EED50286 | EQ963480 |
| *Bulgaria inquinans* | AY789345 | DQ470960 | DQ470910 | DQ471079 |
| *Candida albicans* | AF217609 | FJ627956 | NW139465 | AACQ01000177 |
| *Coccomyces dentatus* | DQ491499 | AY544657 | DQ247789 | DQ497605 |
| *Cryptococcus neoformans* | AF444326 | L14067 | XM_570204 | U81803 |
| *Dermatocarpon miniatum* | DQ782837 | AY584644 | DQ782863 | DQ782893 |
| *Dothidea insculpta* | AF027764 | DQ247802 | DQ247792 | DQ471081 |
| *Geoglossum nigritum* | DQ491490 | AY544650 | DQ470879 | DQ471044 |
| *Lecanora hybocarpa* | DQ782849 | DQ782910 | DQ782871 | DQ782901 |
| *Leotia lubrica* | DQ491484 | AY544644 | DQ470876 | DQ471041 |
| *Magnaporthe grisea* | GU073120 | DQ493955 | NW001798721 | NW001798829 |
| *Meria laricis* | U92299 | DQ470954 | DQ470904 | DQ842026 |
| *Morchella elata* | GQ249383 | HM756732 | AF107810 | HM756737 |
| *Morchella esculenta* | AJ543738 | AJ698465 | DQ470880 | DQ471046 |
| *Neurospora crassa* | JN628081 | AF286411 | XM_324476 | XM_959775 |
| *Orbilia auricolor* | DQ491512 | DQ470953 | DQ470903 | DQ471072 |
| *Orbilia vinosa* | DQ491511 | DQ470952 |  | DQ471071 |
| *Phaeosphaeria nodorum* | GQ922523 | EU754175 | DQ499809 | GU456285 |
| *Pleopsidium gobiense* | HQ650723 | DQ883698 | DQ525452 | DQ883804 |
| *Rhizopus oryzae* | JN943054 | JN938902 | JN993503 | AB512252 |
| *Roccellographa cretacea* | AF138825 | DQ883696 | DQ883713 | DQ883733 |
| *Saccharomyces cerevisiae* | JF715201 | GQ222350 | NC001147 | NC001134 |
| *Schismatomma decolora* | AY548808 | AY548815 | DQ883715 | DQ883725 |
| *Schizosaccharomyces pombe* | EU916982 | EU916982 | NC003424 | NM001022750 |
| *Sclerotinia sclerotiorum* | JQ618848 | DQ470965 | DQ470916 | DQ471086 |
| *Taphrina deformans* | AF492093 | DQ470973 | DQ470927 | DQ471097 |
| *Trichoglossum hirsutum* | DQ491494 | AY544653 | DQ470881 | DQ471049 |
| *Tryblidiopsis pinastri* | FN868463 | HM140573 | DQ470935 | DQ471106 |
| *Xylaria hypoxylon* | DQ491487 | AY544648 | DQ470878 | DQ471042 |
