# Supplementary material for: Multigene Molecular Phylogeny and Biogeographic Diversification of the Earth Tongue Fungi in the Genera Cudonia and Spathularia (Rhytismatales, Ascomycota)
Source: PLoS One. 2014 Aug 1;9(8):e103457. doi: 10.1371/journal.pone.0103457 (PMC4118880; doi:10.1371/journal.pone.0103457)
Supplement: Table S4 — Evolutionary divergence of ITS sequence pairs within and between putative species of Cudonia and Spathularia. (DOC) [file pone.0103457.s009.doc]

| **Table S4. Evolutionary divergence of ITS sequence pairs within and between putative species of *Cudonia* and *Spathularia*** | | | | | | | | | | | | | | | | | | | | | | |
| --- | --- | --- | --- | --- | --- | --- | --- | --- | --- | --- | --- | --- | --- | --- | --- | --- | --- | --- | --- | --- | --- | --- |
| Species recognized | | Interspecific variations | | | |  |  |  |  |  |  |  |  |  |  |  |  |  |  |  |  | Intraspecific variations |
| *C_sichuanensis* |  | |  |  |  |  |  |  |  |  |  |  |  |  |  |  |  |  |  |  |  | 0.000 |
| C_sp9 | 0.049 | |  |  |  |  |  |  |  |  |  |  |  |  |  |  |  |  |  |  |  | n/c |
| C_sp15 | 0.036 | | 0.039 |  |  |  |  |  |  |  |  |  |  |  |  |  |  |  |  |  |  | n/c |
| C_sp14 | 0.033 | | 0.042 | 0.013 |  |  |  |  |  |  |  |  |  |  |  |  |  |  |  |  |  | n/c |
| C_sp4 | 0.031 | | 0.044 | 0.015 | 0.008 |  |  |  |  |  |  |  |  |  |  |  |  |  |  |  |  | 0.000 |
| C_sp13 | 0.037 | | 0.046 | 0.019 | 0.009 | 0.009 |  |  |  |  |  |  |  |  |  |  |  |  |  |  |  | 0.002 |
| C_sp8 | 0.036 | | 0.041 | 0.031 | 0.033 | 0.031 | 0.035 |  |  |  |  |  |  |  |  |  |  |  |  |  |  | n/c |
| C_sp12 | 0.033 | | 0.044 | 0.028 | 0.031 | 0.028 | 0.022 | 0.026 |  |  |  |  |  |  |  |  |  |  |  |  |  | 0.000 |
| C_sp6 | 0.064 | | 0.051 | 0.051 | 0.059 | 0.056 | 0.061 | 0.045 | 0.054 |  |  |  |  |  |  |  |  |  |  |  |  | 0.003 |
| *C_sp1* | 0.033 | | 0.036 | 0.023 | 0.025 | 0.023 | 0.027 | 0.025 | 0.018 | 0.040 |  |  |  |  |  |  |  |  |  |  |  | 0.000 |
| C_sp5 | 0.044 | | 0.047 | 0.039 | 0.041 | 0.033 | 0.043 | 0.036 | 0.034 | 0.048 | 0.015 |  |  |  |  |  |  |  |  |  |  | n/c |
| C_sp2 | 0.049 | | 0.052 | 0.033 | 0.036 | 0.028 | 0.037 | 0.041 | 0.039 | 0.051 | 0.020 | 0.020 |  |  |  |  |  |  |  |  |  | 0.000 |
| *C_lutea* | 0.046 | | 0.033 | 0.041 | 0.044 | 0.036 | 0.045 | 0.038 | 0.036 | 0.048 | 0.023 | 0.023 | 0.028 |  |  |  |  |  |  |  |  | 0.000 |
| C_sp7 | 0.041 | | 0.039 | 0.031 | 0.033 | 0.031 | 0.035 | 0.033 | 0.026 | 0.046 | 0.013 | 0.023 | 0.028 | 0.020 |  |  |  |  |  |  |  | n/c |
| *C_sp11* | 0.038 | | 0.046 | 0.027 | 0.030 | 0.027 | 0.021 | 0.030 | 0.012 | 0.054 | 0.014 | 0.026 | 0.032 | 0.029 | 0.013 |  |  |  |  |  |  | 0.002 |
| *C_circinanas* | 0.036 | | 0.045 | 0.026 | 0.029 | 0.026 | 0.019 | 0.029 | 0.011 | 0.052 | 0.013 | 0.023 | 0.029 | 0.026 | 0.015 | 0.003 |  |  |  |  |  | 0.001 |
| C_sp3 | 0.047 | | 0.054 | 0.032 | 0.034 | 0.032 | 0.025 | 0.029 | 0.021 | 0.050 | 0.022 | 0.032 | 0.032 | 0.035 | 0.024 | 0.014 | 0.011 |  |  |  |  | 0.001 |
| C_confusa | 0.029 | | 0.041 | 0.024 | 0.026 | 0.022 | 0.020 | 0.030 | 0.015 | 0.053 | 0.022 | 0.037 | 0.041 | 0.037 | 0.030 | 0.019 | 0.018 | 0.028 |  |  |  | 0.004 |
| C-constrictospora | 0.055 | | 0.055 | 0.044 | 0.047 | 0.044 | 0.040 | 0.050 | 0.039 | 0.068 | 0.039 | 0.055 | 0.061 | 0.055 | 0.047 | 0.038 | 0.037 | 0.047 | 0.032 |  |  | n/c |
| C_sp10 | 0.052 | | 0.018 | 0.036 | 0.044 | 0.042 | 0.035 | 0.036 | 0.031 | 0.059 | 0.039 | 0.050 | 0.055 | 0.042 | 0.047 | 0.033 | 0.031 | 0.040 | 0.030 | 0.047 |  | n/c |
| *C_monticola* | 0.142 | | 0.161 | 0.152 | 0.153 | 0.147 | 0.143 | 0.151 | 0.145 | 0.169 | 0.155 | 0.155 | 0.165 | 0.158 | 0.161 | 0.147 | 0.146 | 0.149 | 0.138 | 0.158 | 0.144 | 0.005 |
|  |  | |  |  |  |  |  |  |  |  |  |  |  |  |  |  |  |  |  |  |  |  |
| S. sp6 |  | |  |  |  |  |  |  |  |  |  |  |  |  |  |  |  |  |  |  |  | 0.001 |
| S. sp | 0.072 | |  |  |  |  |  |  |  |  |  |  |  |  |  |  |  |  |  |  |  | 0 |
| *S. rufa* | 0.021 | | 0.059 |  |  |  |  |  |  |  |  |  |  |  |  |  |  |  |  |  |  | 0.003 |
| S. sp5 | 0.037 | | 0.061 | 0.027 |  |  |  |  |  |  |  |  |  |  |  |  |  |  |  |  |  | 0 |
| S. sp7 | 0.032 | | 0.061 | 0.022 | 0.018 |  |  |  |  |  |  |  |  |  |  |  |  |  |  |  |  | n/c |
| S. sp4 | 0.061 | | 0.063 | 0.054 | 0.046 | 0.033 |  |  |  |  |  |  |  |  |  |  |  |  |  |  |  | 0 |
| *S. velutipes* | 0.065 | | 0.069 | 0.062 | 0.057 | 0.055 | 0.055 |  |  |  |  |  |  |  |  |  |  |  |  |  |  | 0 |
| S. sp3 | 0.056 | | 0.061 | 0.046 | 0.041 | 0.036 | 0.041 | 0.033 |  |  |  |  |  |  |  |  |  |  |  |  |  | n/c |
| S. sp1 | 0.068 | | 0.084 | 0.063 | 0.058 | 0.049 | 0.057 | 0.055 | 0.05 |  |  |  |  |  |  |  |  |  |  |  |  | 0 |
| S. sp2 | 0.065 | | 0.081 | 0.06 | 0.06 | 0.052 | 0.06 | 0.052 | 0.042 | 0.01 |  |  |  |  |  |  |  |  |  |  |  | 0 |
| *S. flavida* | 0.078 | | 0.087 | 0.069 | 0.07 | 0.062 | 0.07 | 0.065 | 0.051 | 0.027 | 0.022 |  |  |  |  |  |  |  |  |  |  | 0.003 |
